# Supplementary material for: The chimeric aptamer axl-miR-214sponge inhibits breast cancer and melanoma dissemination
Source: Mol Ther. 2025 Jul 31;33(11):5804–16. doi: 10.1016/j.ymthe.2025.07.039 (PMC12628062; doi:10.1016/j.ymthe.2025.07.039)
Supplement: Document S1. Figures S1–S8 [file mmc1.pdf]

YMTHE, Volume *33*

Supplemental Information

The chimeric aptamer axl-miR-214sponge inhibits breast cancer and melanoma dissemination

Lorena Quirico, Sabrina Rizzolio, Sofia Bertone, Priscila D.R. Cirillo, Aurora Savino, Nicoletta Vitale, Silvia Catuogno, Carla L. Esposito, Michael B. Stadler, Paola Defilippi, Vittorio de Franciscis, Francesca Orso, and Daniela Taverna

Figure S1

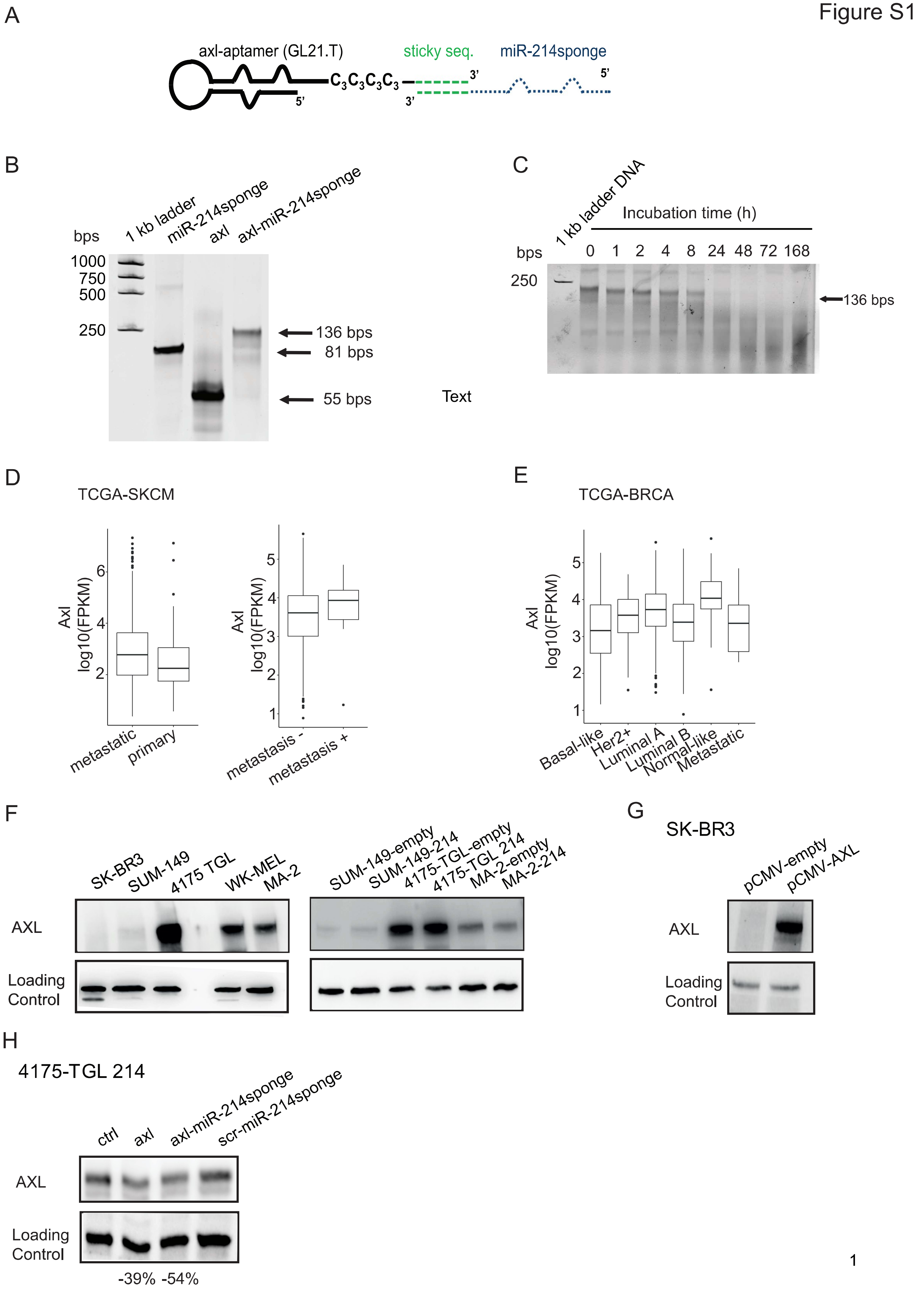
A
axl-aptamer (GL21.T)
sticky seq.
miR-214sponge
C3C3C3C3
3'
5'
5'
3'
B
bps
1 kb ladder
miR-214sponge
axl
axl-miR-214sponge
1000
750
500
250
136 bps
81 bps
55 bps
Text
C
1 kb ladder DNA
Incubation time (h)
bps
0 1 2 4 8 24 48 72 168
250
136 bps
D
TCGA-SKCM
Axl
log10(FPKM)
6
4
2
metastatic
primary
5
4
3
2
1
metastasis -
metastasis +
E
TCGA-BRCA
Axl
log10(FPKM)
5
4
3
2
1
Basal-like
Her2+
Luminal A
Luminal B
Normal-like
Metastatic
F
SK-BR3
SUM-149
4175 TGL
WK-MEL
MA-2
SUM-149-empty
SUM-149-214
4175-TGL-empty
4175-TGL 214
MA-2-empty
MA-2-214
AXL
Loading Control
G
SK-BR3
pCMV-empty
pCMV-AXL
AXL
Loading Control
H
4175-TGL 214
ctrl
axl
axl-miR-214sponge
scr-miR-214sponge
AXL
Loading Control
-39% -54%

Figure S1–Axl-miR-214sponge chimeric aptamer generation and axl expression. (A) Schematic representation of axl-miR-214sponge chimeric aptamer. (B) Representative polyacrylamide gel electrophoresis to verify the proper chimera formation. The 136 bps band corresponds to the axl-miR-214sponge conjugate, the 81 bps band shows the miR-214sponge fragment, the 55 bps coincides with the axl aptamer alone. (C) A representative picture of non-denaturing polyacrylamide gel electrophoresis showing the stability of axl-miR-214sponge chimeric aptamer (4 μM) in 80% human serum for the indicated time (hours=h). (D) axl expression (mRNA) in human metastatic (n=368) and primary (n=103) melanoma samples or in primary melanomas from patients with (n=3) or without (n=98) relapse (metastasis +/-) as obtained from TCGA-SKCM datasets. (E) axl expression (mRNA) in human Basal-like (n =190), Her2+ (n=82), Luminal A (n =562) or B (n =209), Normal-like (n =40) or metastatic (n =7) breast cancer samples as obtained from TCGA-BRCA datasets. TCGA data were downloaded through the TCGAbiolinks. Expression data are presented as log10(FPKM) with error bars representing standard deviations. (F) Western Blot (WB) analysis of axl expression in native or empty or miR-214 overexpressing (214) human breast cancer or melanoma SK-BR3, SUM-149, 4175-TGL, WK-MEL 214, MA-2 cell lines. GAPDH was used as loading control. (G) WB analysis for axl expression in SK-BR3 cells transfected with pCMV-AXL or pCMV-empty expression vectors. GAPDH was used as loading control. (H) WB analysis for axl expression in miR-214 overexpressing 4175-TGL cells following treatments with PBS control (ctrl) or axl or axl-miR-214sponge or scr-miR-214sponge (scramble) aptamer solutions for 48 hours. Protein modulations were calculated relative to ctrl and normalized on loading controls (vinculin) and expressed as percentages (%). (B-C) 1kb ladder DNA was used as referral for molecular weights. (B-H) Three independent experiments were performed, and representative results are shown. FPKM= Fragments Per Kilobase per Million mapped fragments.

Figure S2

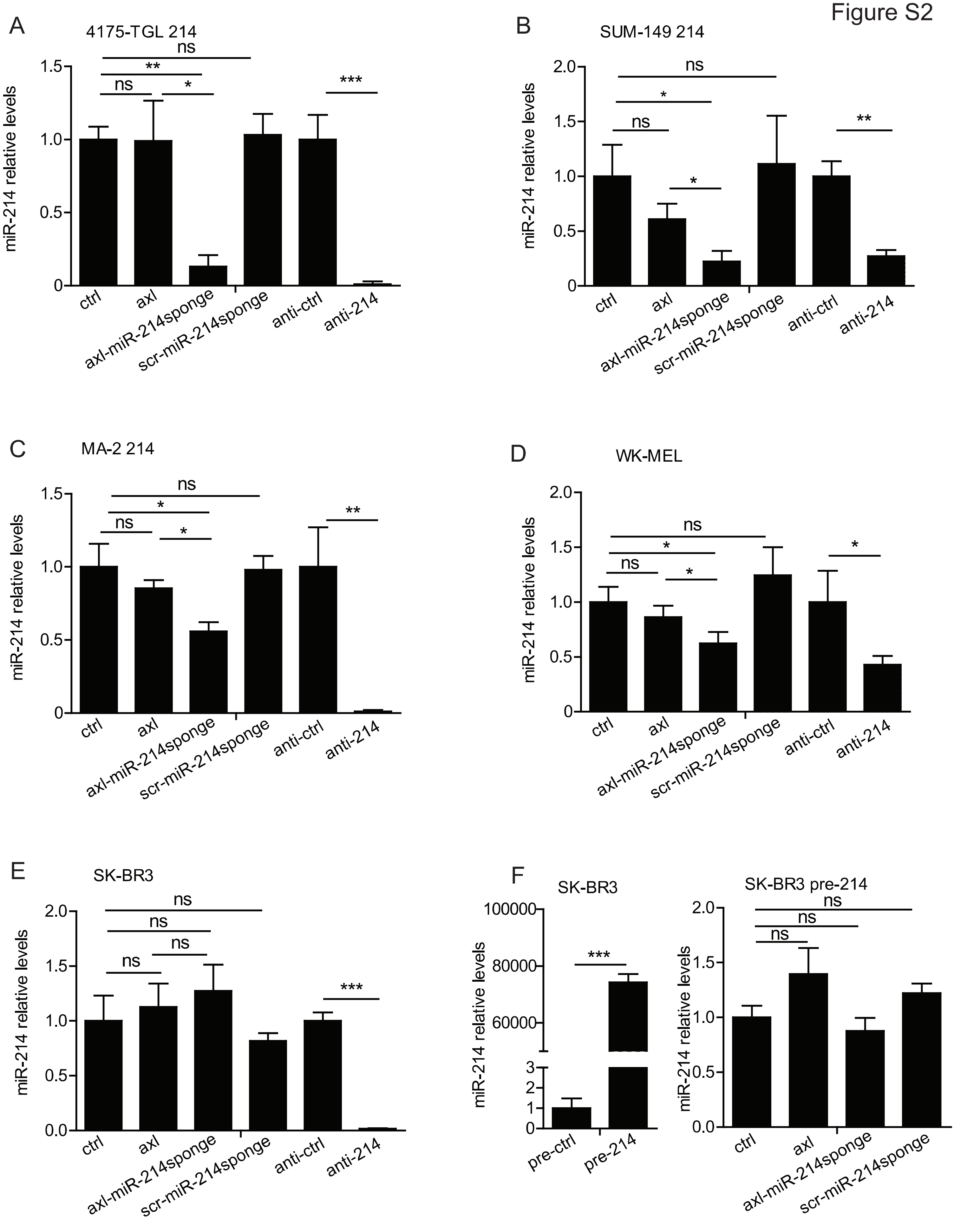

A
4175-TGL 214
miR-214 relative levels
1.5
1.0
0.5
0
ns
**
ns
*
***
ctrl
axl
axl-miR-214sponge
scr-miR-214sponge
anti-ctrl
anti-214
B
SUM-149 214
miR-214 relative levels
2.0
1.5
1.0
0.5
0
ns
*
ns
*
**
ctrl
axl
axl-miR-214sponge
scr-miR-214sponge
anti-ctrl
anti-214
C
MA-2 214
miR-214 relative levels
1.5
1.0
0.5
0
ns
*
ns
*
**
ctrl
axl
axl-miR-214sponge
scr-miR-214sponge
anti-ctrl
anti-214
D
WK-MEL
miR-214 relative levels
2.0
1.5
1.0
0.5
0
ns
*
ns
*
*
ctrl
axl
axl-miR-214sponge
scr-miR-214sponge
anti-ctrl
anti-214
E
SK-BR3
miR-214 relative levels
2.0
1.5
1.0
0.5
0.0
ns
ns
ns
ns
***
ctrl
axl
axl-miR-214sponge
scr-miR-214sponge
anti-ctrl
anti-214
F
SK-BR3
miR-214 relative levels
100000
80000
60000
3
2
1
0
***
pre-ctrl
pre-214
SK-BR3 pre-214
miR-214 relative levels
2.0
1.5
1.0
0.5
0.0
ns
ns
ns
ctrl
axl
axl-miR-214sponge
scr-miR-214sponge

Figure S2 – Axl-miR-214sponge chimeric aptamer affects miR-214 expression. (A-F) miR-214 relative expression levels for *axl*-positive miR-214 overexpressing 4175-TGL, SUM-149 or MA-2 cells or WK-MEL or *axl*-negative SK-BR3 cells after treatments with control (ctrl) solution, axl aptamer alone, axl-miR-214sponge or scr-miR-214sponge conjugates, evaluated by qRT-PCR analysis. Transfection with anti-miR-214 (anti-214, A-E) or pre-miR-214 (pre-214, F, left panel) or their relative controls (anti-ctrl or pre-ctrl) were also performed and miR-214 expression levels analyzed by qRT-PCR analysis. Results are shown as fold changes (mean ± SD) relative to controls, normalized on U44 or U6 small nucleolar RNA levels. Three independent experiments in triplicate were performed and a representative one is shown. ns= not significant, * $p < 0.05$, ** $p< 0.01$, *** $p< 0.001$. SD= Standard Deviation.

Figure S3

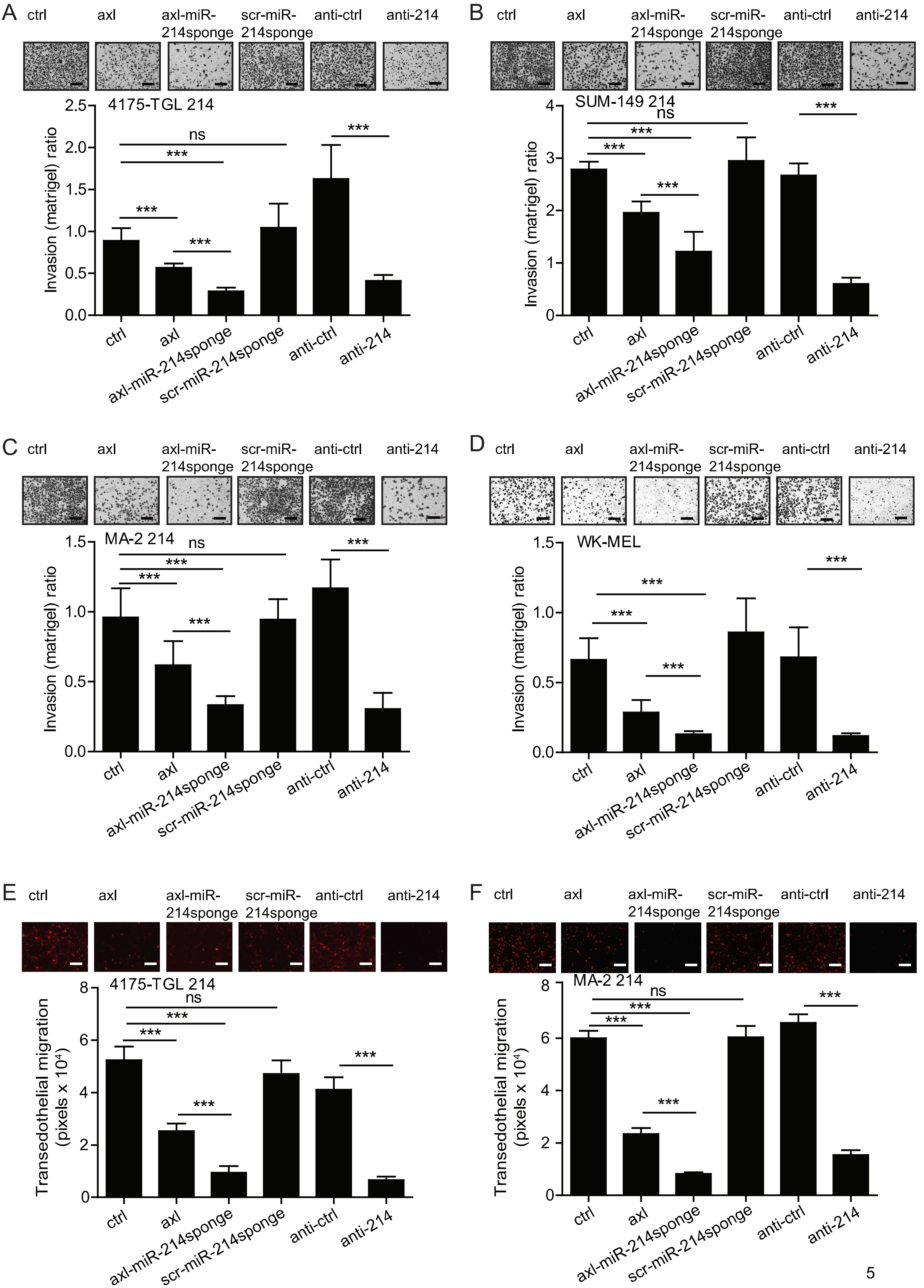
A
ctrl
axl
axl-miR-214sponge
scr-miR-214sponge
anti-ctrl
anti-214
4175-TGL 214
Invasion (matrigel) ratio
0.0
0.5
1.0
1.5
2.0
2.5
ns
***
B
SUM-149 214
0
1
2
3
4
C
MA-2 214
D
WK-MEL
E
F
Transedothelial migration (pixels x 10⁴)
0
2
4
6
8

Figure S3 - Axl-miR-214sponge chimeric aptamer impairs cancer cell invasion.

(A-F) Invasion through Matrigel (A-D) or through a HUVEC monolayer (E-F) assays for *axl*-positive miR-214 overexpressing 4175-TGL (A, E), SUM-149 (B), MA-2 (C, F) or not-engineered WK-MEL (D) cells treated with ctrl solution, axl aptamer alone or axl-miR-214sponge or scr-miR-214sponge conjugates. Transfections with anti-miR-214 (anti-214) and its relative control (anti-ctrl) were also performed. Top: representative photographs of invaded/transmigrated cells. Bottom: graphs of results expressed as (A-D) ratio of mean ± SEM of the area covered by invaded versus plated cancer cells or as (E-F) mean±SEM of the area (pixels) covered by transmigrated cells. At least two independent experiments (in triplicate) were performed and representative results are shown. ns = not significant; *** $p < 0.001$; SEM = Standard Error of Mean; scale bar = 50 μm.

Figure S4

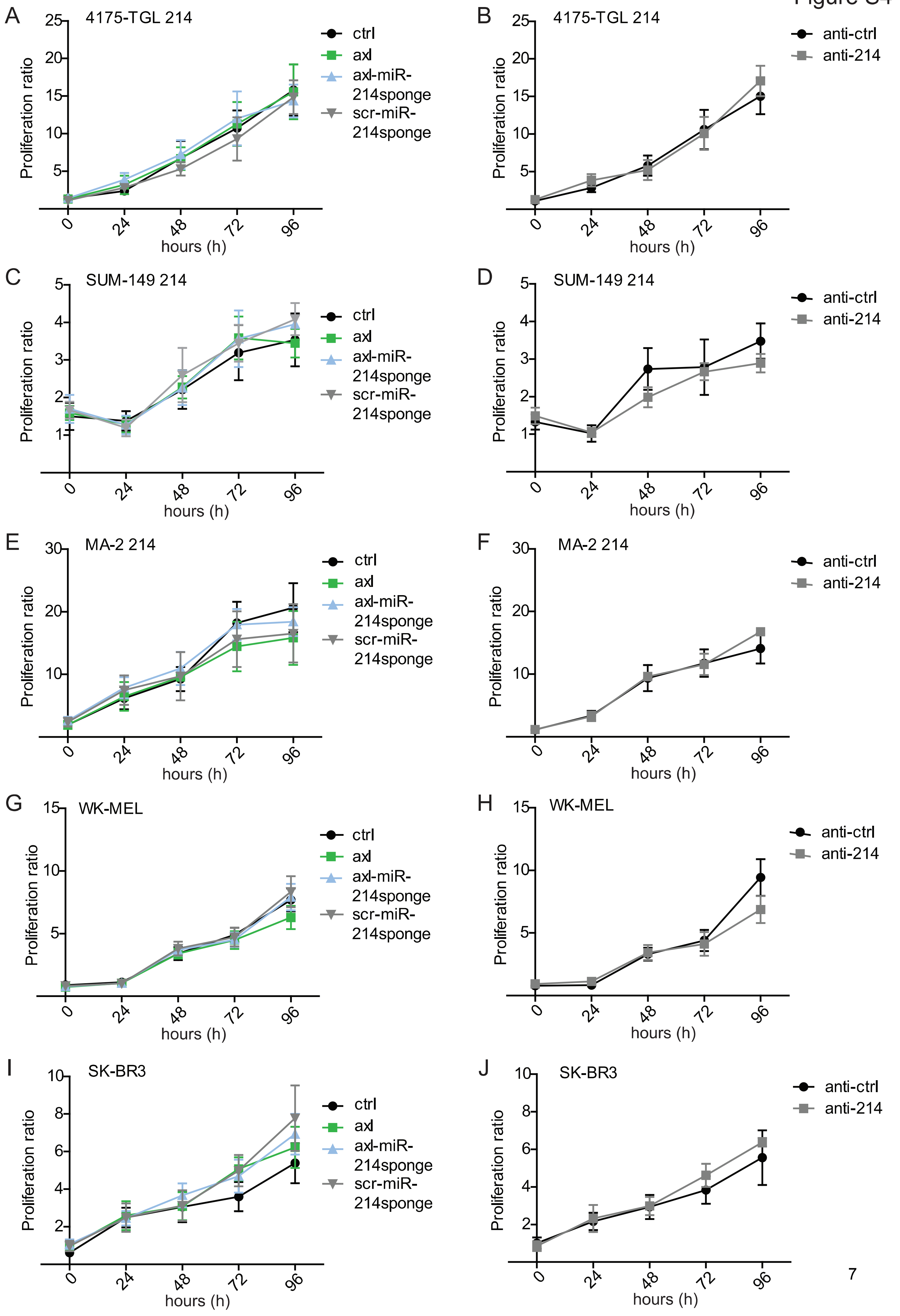

A
4175-TGL 214
Proliferation ratio
hours (h)
ctrl
axl
axl-miR-214sponge
scr-miR-214sponge
B
4175-TGL 214
anti-ctrl
anti-214
C
SUM-149 214
D
SUM-149 214
E
MA-2 214
F
MA-2 214
G
WK-MEL
H
WK-MEL
I
SK-BR3
J
SK-BR3

Figure S4– Axl-miR-214sponge chimeric aptamer does not affect *in vitro* proliferation. (A-J) Proliferation for *axl*-positive miR-214 overexpressing 4175-TGL (A-B), SUM-149 (C-D), MA-2 (E-F) or not-engineered WK-MEL (G-H) or *axl*-negative SK-BR3 (I-J) cells treated with ctrl solution, axl aptamer alone or axl-miR-214sponge or scr-miR-214sponge conjugates. Transfections with anti-miR-214 (anti-214) and its relative control (anti-ctrl) were also performed. Results are represented as mean±SD of the proliferation *ratio* versus plated cells, measured by optical density at 0-96h. At least 2 independent experiments (with triplicates) were performed and representative results are shown. SD = Standard Deviation.

Figure S5

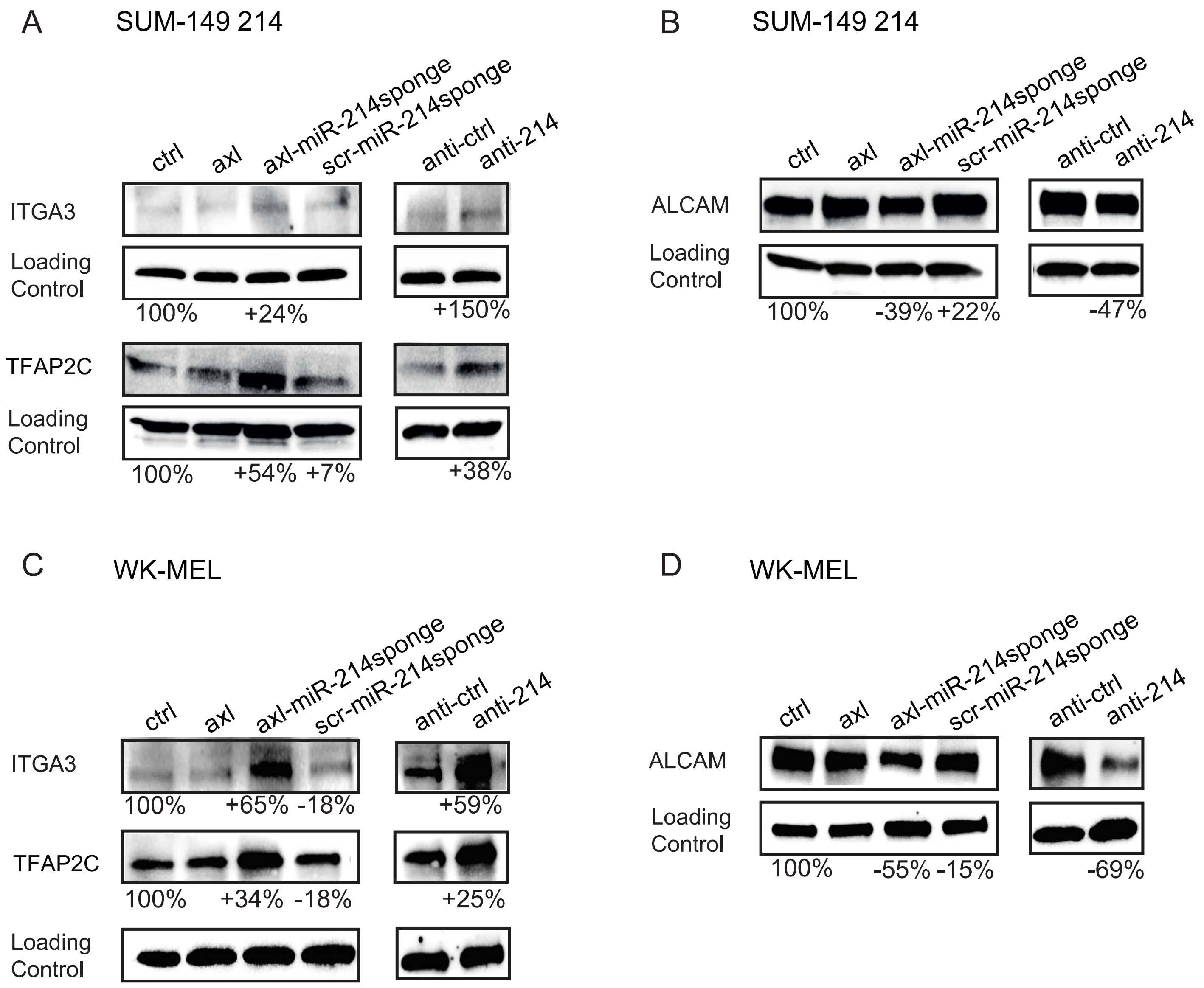
A
SUM-149 214
ctrl
axl
axl-miR-214sponge
scr-miR-214sponge
anti-ctrl
anti-214
ITGA3
Loading Control
100%
+24%
+150%
TFAP2C
Loading Control
100%
+54%
+7%
+38%
B
SUM-149 214
ctrl
axl
axl-miR-214sponge
scr-miR-214sponge
anti-ctrl
anti-214
ALCAM
Loading Control
100%
-39%
+22%
-47%
C
WK-MEL
ctrl
axl
axl-miR-214sponge
scr-miR-214sponge
anti-ctrl
anti-214
ITGA3
100%
+65%
-18%
+59%
TFAP2C
100%
+34%
-18%
+25%
Loading Control
D
WK-MEL
ctrl
axl
axl-miR-214sponge
scr-miR-214sponge
anti-ctrl
anti-214
ALCAM
Loading Control
100%
-55%
-15%
-69%

Figure S5– Axl-miR-214sponge chimeric aptamer affects miR-214 direct and indirect targets in *axl*-expressing cancer cells. (A-D) Western blot analysis of miR-214 direct targets ITGA3 and TFAP2C or indirect target ALCAM in *axl*-positive SUM-149 214 (A-B), WK-MEL (C-D) treated with ctrl solution, axl aptamer alone, axl-miR-214sponge or scr-miR-214sponge conjugates. Transfections with anti-miR-214 (anti-214) and its relative control (anti-control) were also performed. After 48 hours proteins were collected and protein modulations calculated relative to axl or anti-control and normalized on loading controls: hsp90 (A, top) or GAPDH (A, bottom; B; C, left; D, right) or vinculin (C, right) and expressed as percentages (%). At least three independent experiments were performed and representative results are shown.

Figure S6

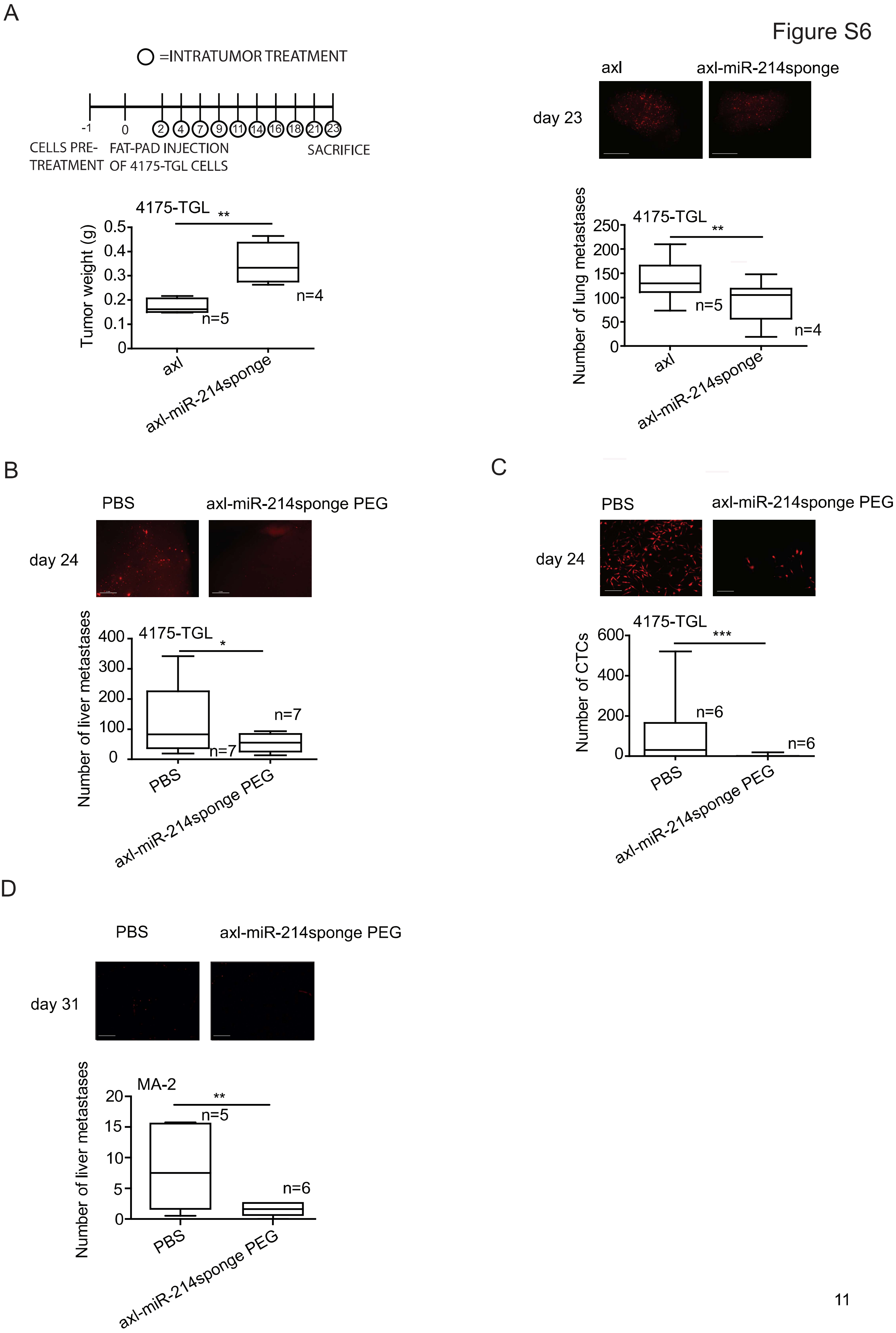
A
=INTRATUMOR TREATMENT
-1
0
2
4
7
9
11
14
16
18
21
23
CELLS PRE-TREATMENT
FAT-PAD INJECTION OF 4175-TGL CELLS
SACRIFICE
4175-TGL
**
Tumor weight (g)
0.5
0.4
0.3
0.2
0.1
0
n=5
n=4
axl
axl-miR-214sponge
axl
axl-miR-214sponge
day 23
4175-TGL
**
Number of lung metastases
250
200
150
100
50
0
n=5
n=4
axl
axl-miR-214sponge
B
PBS
axl-miR-214sponge PEG
day 24
4175-TGL
*
Number of liver metastases
400
300
200
100
0
n=7
n=7
PBS
axl-miR-214sponge PEG
C
PBS
axl-miR-214sponge PEG
day 24
4175-TGL
***
Number of CTCs
600
400
200
0
n=6
n=6
PBS
axl-miR-214sponge PEG
D
PBS
axl-miR-214sponge PEG
day 31
MA-2
**
Number of liver metastases
20
15
10
5
0
n=5
n=6
PBS
axl-miR-214sponge PEG

Figure S6 –Axl-miR-214sponge chimeric aptamer intratumor or systemic treatments reduce breast cancer dissemination in mice. (A) Scheme of the experiment: Red Fluorescent Protein (RFP)-expressing 4175-TGL cells pretreated with axl or axl-miR-214sponge aptamers were injected into the mammary gland fat pad of NOD/SCID/IL2R null mice. Then, axl or axl-miR-214sponge aptamers were administered into the tumors starting from 2 days post-injection (3 treatments/week, 300 pmol in 100 μl, 10 injections in total, as indicated) and primary tumor weight (grams = g) and total number of lung metastases (mean±SEM) were analyzed 23 days post-tumor-cell injections for the indicated number (n) of mice. (B-D) Number of liver metastases (B, D) and Circulating Tumor Cells (CTCs) (C) referring to the experiments presented in Figure 4A-B. Representative images of fluorescent lung or liver metastases or CTCs are shown. ** $p < 0.01$; SEM = Standard Error of Mean; Scale bar=800 μm.

Figure S7

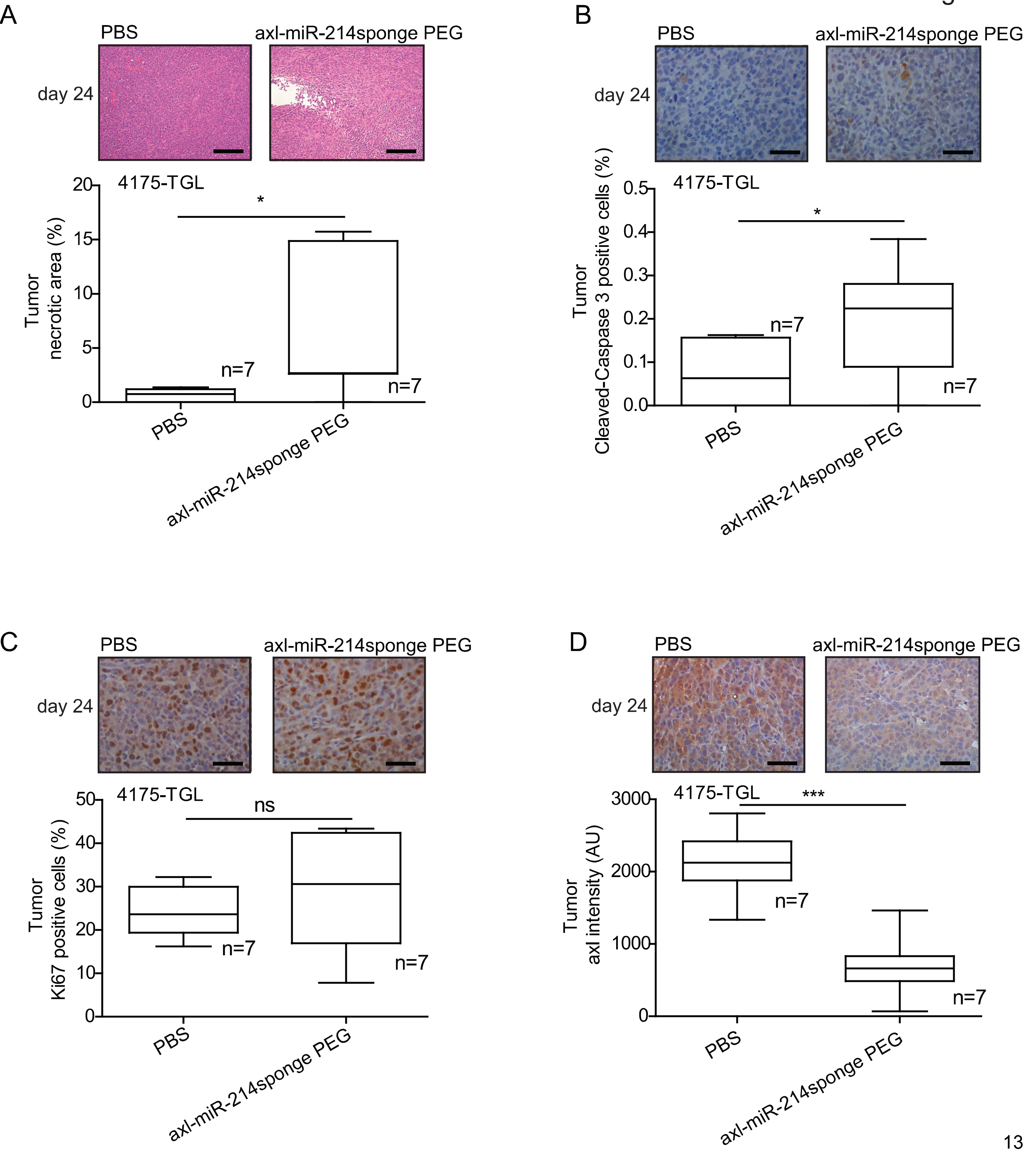

A
PBS
axl-miR-214sponge PEG
day 24
4175-TGL
Tumor necrotic area (%)
n=7
n=7
PBS
axl-miR-214sponge PEG
B
PBS
axl-miR-214sponge PEG
day 24
4175-TGL
Tumor Cleaved-Caspase 3 positive cells (%)
n=7
n=7
PBS
axl-miR-214sponge PEG
C
PBS
axl-miR-214sponge PEG
day 24
4175-TGL
ns
Tumor Ki67 positive cells (%)
n=7
n=7
PBS
axl-miR-214sponge PEG
D
PBS
axl-miR-214sponge PEG
day 24
4175-TGL
***
Tumor axl intensity (AU)
n=7
n=7
PBS
axl-miR-214sponge PEG

Figure S7 - Axl-miR-214sponge chimeric aptamer induces necrosis and apoptosis in breast cancer xenotransplants. (A-D) FFPE sections of 4175-TGL cell-derived primary tumors treated as in Figure 4B were stained with (A) H&E and necrotic areas evaluated: representative images are shown on top of plots presenting the percentage (%) of necrotic versus total areas shown as mean±SEM for the indicated number (n) of mice (10 fields/each mouse). Primary tumors were also stained for (B) Cleaved Caspase-3, (C) Ki67 or (D) *axl* and nuclei were counterstained with Hematoxylin (blue). Representative pictures are shown on top of plots presenting the percentage (%) of positive versus total cells (A-C) or staining intensity (D) shown as mean±SEM for the indicated number (n) of mice (10 fields/each mouse). IHC = immunohistochemistry; H&E = Hematoxilyin & Eosin; FFPE: Formalin-Fixed, Paraffin Embedded; ns = not significant; * $p < 0.05$; *** $p < 0.001$; SEM = Standard Error of Mean; scale bar = 100 µm (A) or 25 µm (B-D).

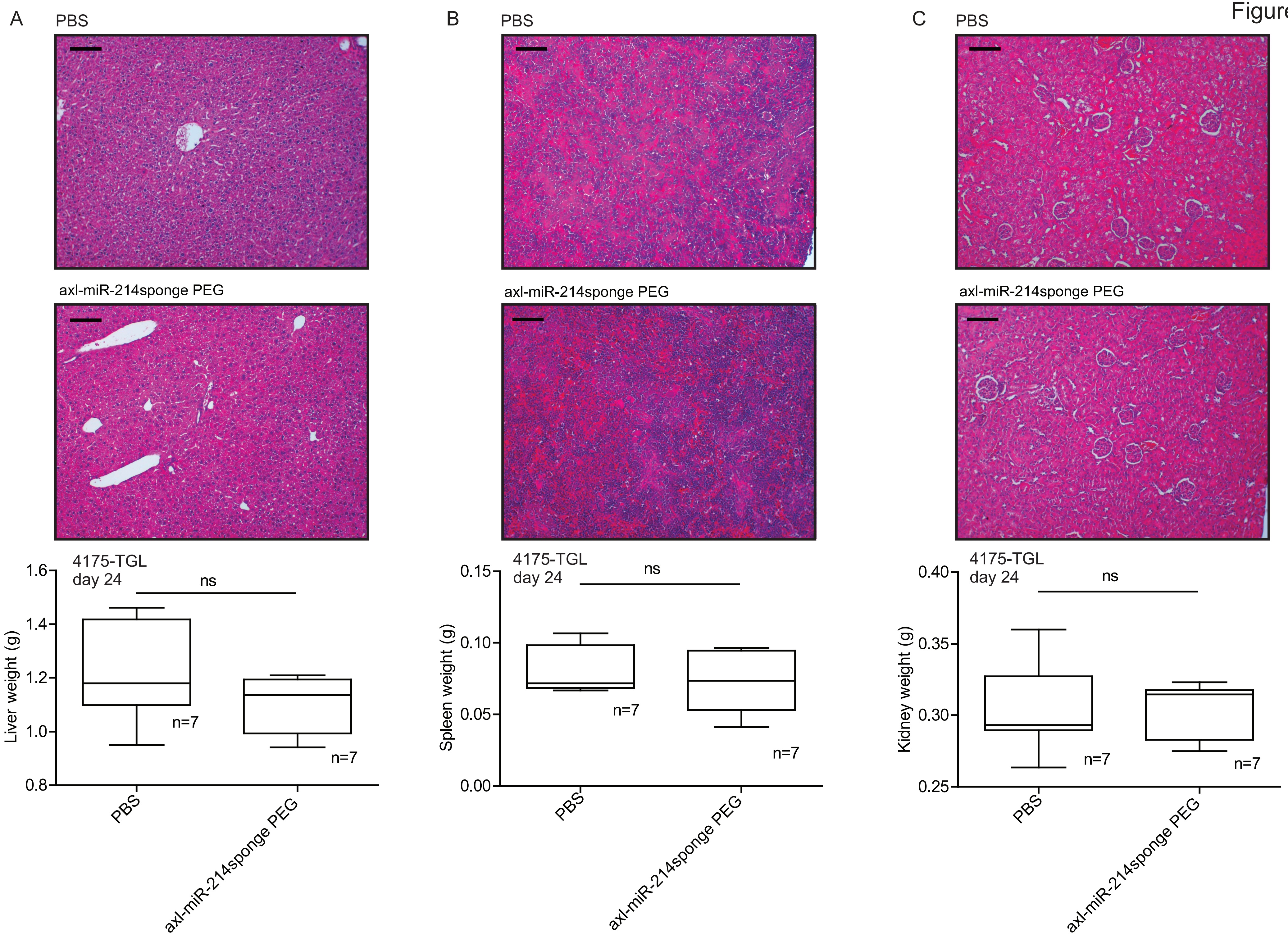
A
PBS
axl-miR-214sponge PEG
4175-TGL
day 24
ns
Liver weight (g)
1.6
1.4
1.2
1.0
0.8
n=7
n=7
PBS
axl-miR-214sponge PEG
B
PBS
axl-miR-214sponge PEG
4175-TGL
day 24
ns
Spleen weight (g)
0.15
0.10
0.05
0.00
n=7
n=7
PBS
axl-miR-214sponge PEG
C
PBS
axl-miR-214sponge PEG
4175-TGL
day 24
ns
Kidney weight (g)
0.40
0.35
0.30
0.25
n=7
n=7
PBS
axl-miR-214sponge PEG

Figure S8– Axl-miR-214sponge chimeric aptamer is not toxic for mice when delivered systemically. (A-C) Mice were injected with Red Fluorescent Protein (RFP)-expressing 4175-TGL cells and treated as in Figure 4B. The weight of liver (A), spleen (B) and kidneys (C) was evaluated at day 24 (final point) and sections stained with H&E. Representative pictures of organs (scale bar=100 μm) are shown on top of plots representing the mean±SEM of weights for the indicated number (n) of mice. ns = not significant; SEM= Standard Error of Mean; H&E = Hematoxilyn & Eosin; g = grams.
