# Supplementary material for: The chimeric aptamer axl-miR-214sponge inhibits breast cancer and melanoma dissemination
Source: Mol Ther. 2025 Jul 31;33(11):5804–16. doi: 10.1016/j.ymthe.2025.07.039 (PMC12628062; doi:10.1016/j.ymthe.2025.07.039)
Supplement: Document S2. Article plus supplemental information [file mmc2.pdf]

Molecular Therapy
Original Article

The chimeric aptamer axl-miR-214sponge inhibits breast cancer and melanoma dissemination

Lorena Quirico,[1,2,10] Sabrina Rizzolio,[1,2,10] Sofia Bertone,[1,2,8] Priscila D.R. Cirillo,[1,2,9] Aurora Savino,[3] Nicoletta Vitale,[1,2] Silvia Catuogno,[4] Carla L. Esposito,[4] Michael B. Stadler,[5] Paola Defilippi,[1,2] Vittorio de Franciscis,[6] Francesca Orso,[1,2,7,11] and Daniela Taverna[1,2,11]

[1]Molecular Biotechnology Center (MBC) "Guido Tarone", 10126 Torino, Italy; [2]Department of Molecular Biotechnology and Health Sciences, University of Torino, 10126 Torino, Italy; [3]Human Technopole, 20157 Milano, Italy; [4]Institute of Endocrinology and Experimental Oncology, CNR, 80131 Napoli, Italy; [5]Friedrich Miescher Institute and Swiss Institute of Bioinformatics, 4056 Basel, Switzerland; [6]Institute of Genetic and Biomedical Research (IRGB), CNR, 20157 Milan, Italy; [7]Department of Translational Medicine, University of Piemonte Orientale, 28100 Novara, Italy

MicroRNAs (miRNAs) are often deregulated in cancer. We previously showed that inhibition of the pro-metastatic miR-214 strongly impairs tumor dissemination. We recently developed a chimeric aptamer, axl-miR-214sponge, including an oligonucleotide sequence able to inhibit miR-214 (miR-214sponge) linked to GL21.T (axl), an aptamer that binds specifically to *axl*, an oncogenic tyrosine kinase receptor abundantly expressed on various malignant melanoma and breast cancer cells. When *axl*-positive but not *axl*-negative cancer cells were treated with axl-miR-214sponge, reduced migration, invasion, and transendothelial migration were observed. In parallel, augmented levels of two miR-214 direct targets, TFAP2C and ITGA3, were seen. Instead, expression of ALCAM, a target of the anti-metastatic miR-148b and downstream effector of miR-214, was found to be decreased. More important, when mice carrying xenotransplants derived from triple-negative breast cancer or melanoma cells were treated *in loco* or systemically with the axl-miR-214sponge conjugates, reduced cancer dissemination was seen, together with increased cell death in primary tumor masses. No toxicity was noted in animals. In summary, our data suggest that axl-miR-214sponge is specific, effective, and safe in blocking *axl*-positive cancer cell spreading. Thus, it represents a promising targeted therapy tool to fight metastasis.

INTRODUCTION

Malignant cancer is often a cause of death worldwide, mostly due to the lack of therapies able to block cancer cell dissemination and metastasis formation in distant organs. In fact, the most commonly used interventions, such as chemotherapy and radiotherapy, are able to control the early disease but often fail in the long term due to the increasing in resistance.[1] In addition, these treatments induce high toxicity with severe side effects, which can seriously affect patients' fitness and quality of life.[2–4] Recently, immunotherapy and targeted therapy have been employed and encouraging improvements in patient life expectancy and well-being have been observed; however, failures and drug resistance prevail too often.[5–7] Therefore, the development of effective and safe treatments able to specifically hit tumor cells and reduce dissemination is still an urgent need.

MicroRNAs (miRNAs) are short non-coding RNAs able to post-transcriptionally regulate gene expression by binding to the 3′ untranslated regions of their target mRNAs. Today, more than 2,000 miRNAs have been identified in human cells, which can control the activity of more than 60% of protein-coding genes.[8] Deregulation of miRNA expression has been observed in several diseases such as neoplasia, multiple sclerosis, systemic lupus erythematosus, neurodegenerative diseases, cardiomyopathies, and myocardial infarction.[9–13] Numerous alterations of miRNA expression have been identified in different kinds of cancers, and the functional relevance of specific miRNAs, together with their molecular interventions, has been emphasized.[14,15]

We previously found that miR-214 is overexpressed in malignant breast cancers and melanomas, that its expression correlates with metastasis formation, and that it was proven to act as a pro-metastatic miRNA.[16] However, no effect on cell proliferation was observed.[16] Regarding the miR-214 molecular mechanism, we observed that it coordinates a pro-metastatic pathway, including transcription factors (i.e., TFAP2C), adhesion molecules (i.e., ALCAM and ITGA3), and the anti-metastatic miR-148b.[17] While we proved that TFAP2C and ITGA3 are miR-214 targets, ALCAM

Received 27 October 2024; accepted 24 July 2025;
https://doi.org/10.1016/j.ymthe.2025.07.039.

[8]Present address: Department of Applied Sciences and Technology, Politecnico di Torino, Turin, Italy
[9]Present address: Diaceutics PLC, Belfast, UK
[10]These authors contributed equally
[11]These authors contributed equally
Correspondence: Paola Defilippi, Molecular Biotechnology Center (MBC) "Guido Tarone", 10126 Torino, Italy.
E-mail: paola.defilippi@unito.it
Correspondence: Daniela Taverna, Molecular Biotechnology Center (MBC) "Guido Tarone", 10126 Torino, Italy.
E-mail: daniela.taverna@unito.it

 © 2025 The Author(s).
Published by Elsevier Inc. on behalf of The American Society of Gene and Cell Therapy.
This is an open access article under the CC BY license (http://creativecommons.org/licenses/by/4.0/).

is indirectly regulated via miR-148b. In fact, when miR-214 overexpression was forced in malignant melanoma or breast cancer cells, miR-148b levels diminished since miR-148b transcription is under the positive control of TFAP2C, and ALCAM is a direct target of miR-148b, as observed previously.[17] In line with this, when miR-214 was depleted, TFAP2C and miR-148b levels increased while ALCAM levels decreased.[17] These data put miR-214 and miR-148b on the same axis, at least partially.

Based on these findings, we evaluated the possibility of using miR-214 and miR-148b as targets for therapy. We proved that combined miR-214 depletion and miR-148b overexpression in malignant melanoma and breast cancer cells could strongly reduce metastasis formation in mice when compared to single miRNA modulations.[18] When we systemically injected stable anti-miR-214 oligos in tumor-bearing mice, we observed reduced extravasation, circulating tumor cells (CTCs), and metastasis formation in lungs and liver.[19] However, with this approach, the anti-miR-214 oligos could reach any cell in the mouse, and not only tumor cells specifically. Based on these results and with the intent to develop tools with a specific delivery to cancer cells, we developed chimeric aptamers for blocking miR-214 activity in cancer cells only. In a recent investigation, we generated a chimeric aptamer, axl-miR-148b (axl-148b), able to bind to *axl*, an oncogenic tyrosine kinase receptor overexpressed on the surface of many malignant cells, and to release miR-148b in the cell to obtain increased miR-148b levels.[20] Several lines of experiments showed that when a specific aptamer for *axl*, GL21.T, was used by itself, the inhibition of metastatic traits could be observed *in vitro*, while impairment of metastasis formation occurred *in vivo*.[20,21] However, when similar experiments were performed employing axl-miR-148b, stronger effects were observed suggesting the benefit of conjugated molecule treatments.[20] Here, we show the capability of another chimeric aptamer, axl-miR-214sponge, to reach *axl*-positive cancer cells specifically, based on the selective recognition of neoplastic cells via the anti-axl aptamer, GL21.T, and to release a sequence able to inhibit miR-214 functions, called miR-214sponge. This conjugate impairs breast cancer and melanoma cell motility *in vitro* in a more pronounced manner than axl aptamer per se and it modulates direct and indirect miR-214 targets. More important, it affects malignancy *in vivo* better than axl aptamer alone. Notably, no effects are detected in cells that do not express *axl* receptor, which underlines its specificity for *axl*-positive cells. These data demonstrate the efficacy of a specific double therapeutic hit (*axl* and miR-214) to counteract metastasis formation.

RESULTS

Axl-miR-214sponge chimeric aptamer generation and characterization

We formerly demonstrated the therapeutic potential of miR-214 downmodulation using anti-miR-214.[18,19] To obtain the specific delivery of a miR-214 inhibitor to miR-214-enriched and *axl*-positive malignant breast cancer and melanoma cells, we generated a chimeric aptamer called axl-miR-214sponge. This compound is composed of two portions: (1) the oligonucleotide GL21.T, an RNA aptamer previously proven to bind to the tyrosine kinase oncogene *axl* with high affinity and specificity[22] (here, simply called axl) and (2) a single-strand DNA sequence able to inhibit miR-214, miR-214sponge, containing two binding sites for miR-214 separated by a 15-nt-linker that confers flexibility and ameliorates miR-214 binding. These portions are joined together via a sticky sequence (Figure S1A). The annealing efficiency was analyzed by non-denaturing polyacrylamide gels for each conjugate preparation, as shown in Figure S1B. The chimeric compound corresponds to the highest band (136 bp), while the middle (81 bp) and the lowest (55 bp) bands coincide, respectively, with miR-214sponge and with the axl aptamer, indicating a proper assembling of axl-miR-214sponge. The conjugate stability was evaluated *in vitro* by incubating it with human serum up to 168 h and analyzing its presence/absence on a non-denaturing polyacrylamide gel (PAGE) at different time points (Figure S1C). axl-miR-214sponge conjugates were stable for about 8 h in the presence of human serum; in fact, degradation was observed at later time points.

Evaluation of *axl* expression was performed on human melanoma and breast cancer samples (primary tumors or metastases) using The Cancer Genome Atlas-skin cutaneous melanoma (TCGA-SKCM) or TCGA-breast invasive carcinoma (BRCA) mRNA datasets and relevant expression was noted for all samples, including primary (from patients with or without relapse) or metastatic specimens and different breast cancer subtypes as in Figures S1D and S1E, thus suggesting that our axl-miR-214sponge conjugates could be largely employed in the clinics. *axl* expression was also analyzed in detail for all cell lines used in this work (SK-BR3, SUM-149, 4175-TGL, WK-MEL, MA-2), engineered or not for miR-214 or AXL overexpression, by western blot (WB) as in Figures S1F and S1G. Glyceraldehyde 3-phosphate dehydrogenase (GAPDH) was used as loading control. All cells, apart from SK-BR3 (used as negative control), were axl-positive. *axl* internalization/degradation was evaluated for miR-214 overexpressing 4175-TGL human breast cancer cells following treatment with either axl alone or axl-miR-214sponge or scr-miR-214sponge (an unrelated/scramble sequence) aptamers for 6 h versus phosphate-buffered saline (PBS)-treated controls (ctrl) by WB analysis (Figure S1H). Reduced *axl* protein levels were noted (expressed as percentages of reduction on control cells) following treatments with axl (axl) and axl-miR-214sponge compared to controls (ctrl or scr-miR-214sponge-treated cells), considering the loading control (vinculin).

Analysis of miR-214 levels via RT-qPCR analysis was then performed on *axl*-expressing breast cancer (4175-TGL 214 or SUM-149 214) or melanoma (MA-2 214 or WK-MEL) cells in culture following 48 h treatments with axl aptamer alone or axl-miR-214sponge conjugate or an unrelated/scramble chimeric complex (scr-miR-214sponge). Cells left untreated (ctrl) were also compared. In parallel, cells were transfected with anti-miR-214 (anti-214) or a control sequence (anti-ctrl) as positive controls. A significant decrease in miR-214 levels was observed in AXL-expressing cells following administration of AXL–miR-214 sponge conjugates,

compared to other treatments. A similar modulation was also seen in cells transfected with anti-miR-214, used here as positive controls based on previous studies[16] (Figures S2A–S2D). Importantly, when the *axl*-negative SK-BR3 breast cancer cells were treated with axl-miR-214sponge, no miR-214 modulation was detected (Figure S2E), while a reduction in miR-214 expression was observed in SK-BR3 cells when transfected with anti-miR-214 sequences, compared to controls, thus indicating that axl-based conjugates work selectively only on *axl*-expressing cells. As additional control, miR-214 levels were increased in SK-BR3 cells by performing transfections with pre-miR-214 (pre-214) and compared to pre-control (pre-ctrl) transfected cells, as in Figure S2F (left panel). Then, these cells were left untreated (ctrl) or treated with axl aptamer alone or axl-miR-214sponge or scr-miR-214sponge and miR-214 levels evaluated by RT-qPCR analysis. Importantly, even when miR-214 levels were increased in these cells, but axl receptors were not present, miR-214 levels did not change following treatments with our conjugates (Figure S2F, right panel).

All these results show the capability of our conjugates to work specifically on axl-positive cells. In fact, miR-214 levels can be reduced by treatments with axl-miR-214sponge conjugates in axl-positive but not in axl-negative cells. Instead, all cells transfected with anti-miR-214 show reduced miR-214 levels.

Axl-miR-214sponge conjugate reduces breast cancer and melanoma cell motility but does not affect *in vitro* proliferation

We previously demonstrated that miR-214 is a pro-metastatic miRNA able to coordinate metastatic traits such as migration, invasion, and metastasis formation.[16] Here, we investigated the ability of axl-miR-214sponge to affect tumor cell migration, invasion through Matrigel, and transendothelial migration through an endothelial cell monolayer (human umbilical vein endothelial cells [HUVECs]), to simulate the extravasation process. Therefore, breast cancer (4175-TGL 214, SUM-149 214, and SK-BR3) or melanoma (MA-2 214 or WK-MEL) cells were left untreated (ctrl) or treated with axl aptamer alone (axl) or with axl-miR-214sponge or scr-miR-214sponge for 48 h. In parallel, cells were transfected with anti-miR-214 (anti-214) or a control sequence (anti-ctrl). Inhibition of cell migration/invasion was observed for *axl*-expressing 4175-TGL 214 (Figures 1A and S3A), SUM-149 214 (Figures 1B and S3B), MA-2 214 (Figures 1C and S3C), or WK-MEL (Figures 1D and S3D) cells, but not for SK-BR3 cells that lack *axl* receptor (Figure 1E), following axl or axl-miR-214sponge treatments compared to controls (ctrl or scr-miR-214sponge). However, when SK-BR3 cells were transfected with the pCMV-AXL vector, *axl* expression was obtained compared to pCMV-empty transfected controls (Figure S1G), and reduced migration was observed as for other *axl*-endogenously expressing cells (Figure 1F). Transendothelial migration was similarly impaired in *axl*-expressing 4175-TGL 214 and MA-2 214 cells (Figures S3E and S3F). As previously seen, axl aptamer alone could block metastatic traits in *axl*-expressing cells[22]; however, a stronger effect was observed for axl-miR-214sponge conjugates, suggesting a combined inhibitory effect on both *axl* and miR-214 activity (Figures 1 and S3). Similar results were observed for the anti-miR-214 (anti-214) transfected cells compared to controls (anti-ctrl) both in cells expressing or not *axl* receptor, since cell transfection does not discriminate cell type. These data suggest that the biological effects obtained rely on axl aptamer-mediated delivery and are specific for cells expressing *axl* on their surface.

To further reinforce our findings, we performed additional experiments in which the strength of anti-miR-214 oligos and axl-miR-214sponge conjugates were compared. Here, *axl*-positive MA-2 cells were transfected with anti-miR-214 (anti-214) or treated with axl-miR-214sponge in miR-214OVER transient conditions (anti-214 + pre-214; axl-miR-214sponge + pre-214). Single transient miR-214 modulations were performed in parallel (transfected: anti-214, pre-214; treated: axl-miR-214sponge). Alternatively, *axl*-negative SK-BR-3 cells were transfected with axl-miR-214sponge and compared to negative controls (scr-miR-214sponge transfected or untreated [ctrl] cells). miR-214 levels were measured (Figures 2A–2C), and migration was evaluated (Figures 2D–2F). These experiments suggest that anti-miR-214 and axl-miR-214sponge, in transfected cells, act similarly in terms of miR-214 reduction and migration inhibition in basic or miR-214 overexpression conditions, independently of axl expression. However, axl-miR-214sponge can be delivered specifically to *axl*-expressing cells (but not to the *axl*-negative counterparts) following aptamer binding to the *axl* receptor and subsequent internalization of the receptor/chimeric aptamer complex, acting as a specifically targeting molecule.

When proliferation was analyzed in cancer cells, no significant changes were detected in treated or transfected cells (Figures S4A–S4J), in line with our previous results[16] showing no effect of miR-214 on proliferation.

All these data indicate the possibility of using axl-miR-214sponge chimeric aptamers to block metastatic traits in AXL-positive cancer cells thanks to specific AXL targeting.

Axl-miR-214sponge chimeric aptamer affects miR-214 direct and indirect targets in *axl*-expressing breast cancer and melanoma cells

miRNA functions are exerted by negative regulations of their target genes; therefore, we analyzed the impact of our conjugates on two formerly identified and well-studied miR-214 direct targets, TFAP2C and integrin α3 (ITGA3).[16] Moreover, since we previously demonstrated that miR-214 coordinates a pathway that includes the blockage of anti-metastatic miR-148b and the release of its direct targets, we also analyzed the effects of the conjugates on an already recognized and well-investigated miR-148b direct target, ALCAM,[17] known to coordinate the metastatic pathway downstream of miR-214. When 4175-TGL 214, SUM-149 214, MA-2 214, or WK-MEL *axl*-expressing cells were treated with axl-miR-214sponge, increased TFAP2C and ITGA3 and decreased

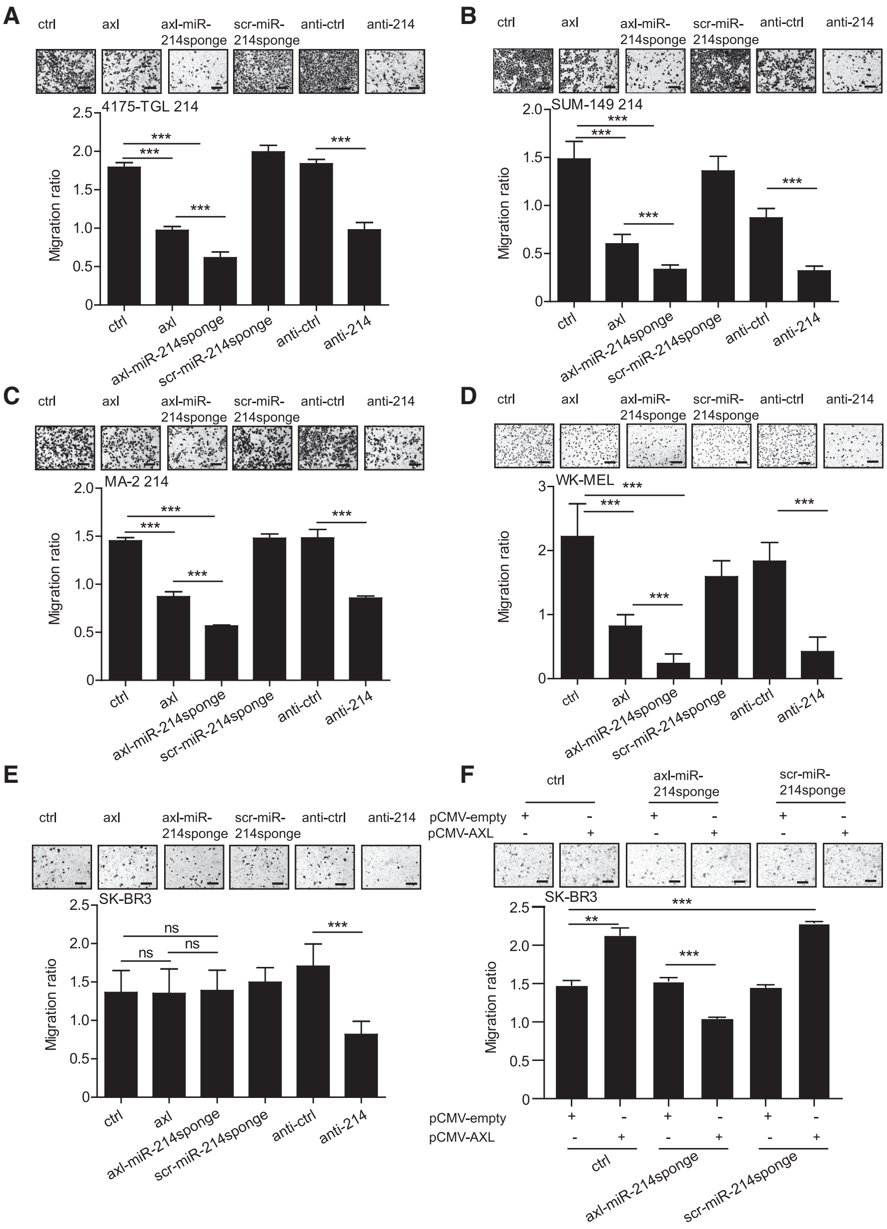

Figure 1. Axl-miR-214sponge conjugate reduces breast cancer and melanoma cell motility

Transwell migration assays for native or miR-214 overexpressing (214) *axl*-positive 4175-TGL (A), SUM-149 (B), MA-2 (C) or WK-MEL (D), *axl*-negative SK-BR3 (E), and *axl* transiently expressing SK-BR3 (F) cells treated with control (ctrl) solution or axl or axl-miR-214sponge or scramble scr-miR-214sponge conjugates. In (F), SKBR-3 cells have been transiently transfected with pCMV-AXL or with pCMV-empty vector and treated with the conjugates or handled as controls. Transfections with anti-miR-214 (anti-214) and its relative negative control (anti-ctrl) were also performed. Top: representative photographs of migrated cells. Bottom: graphs of results expressed as the ratio of mean ± SEM of the area covered by migrated versus plated cancer cells. At least three independent experiments (in triplicate) were performed, and representative results are shown. ns, not significant; *$p < 0.05$; **$p < 0.01$; ***$p < 0.001$; scale bar, 50 μm.

ALCAM protein expression were found compared to ctrl, axl, or scr-miR-214sponge treated cells. Instead, no effect was seen when *axl*-negative SK-BR3 cells were treated with any conjugate, while modulations were observed for all cells following transfections with anti-214 or anti-ctrl, thus underlying the specificity of our chimeric aptamers via *axl* recognition on the cell surface (Figures 3A–3F and S5A–S5D). We can conclude by saying that axl-miR-214sponge is able to inhibit metastatic traits specifically for *axl*-positive cells by affecting direct and indirect miR-214 targets.

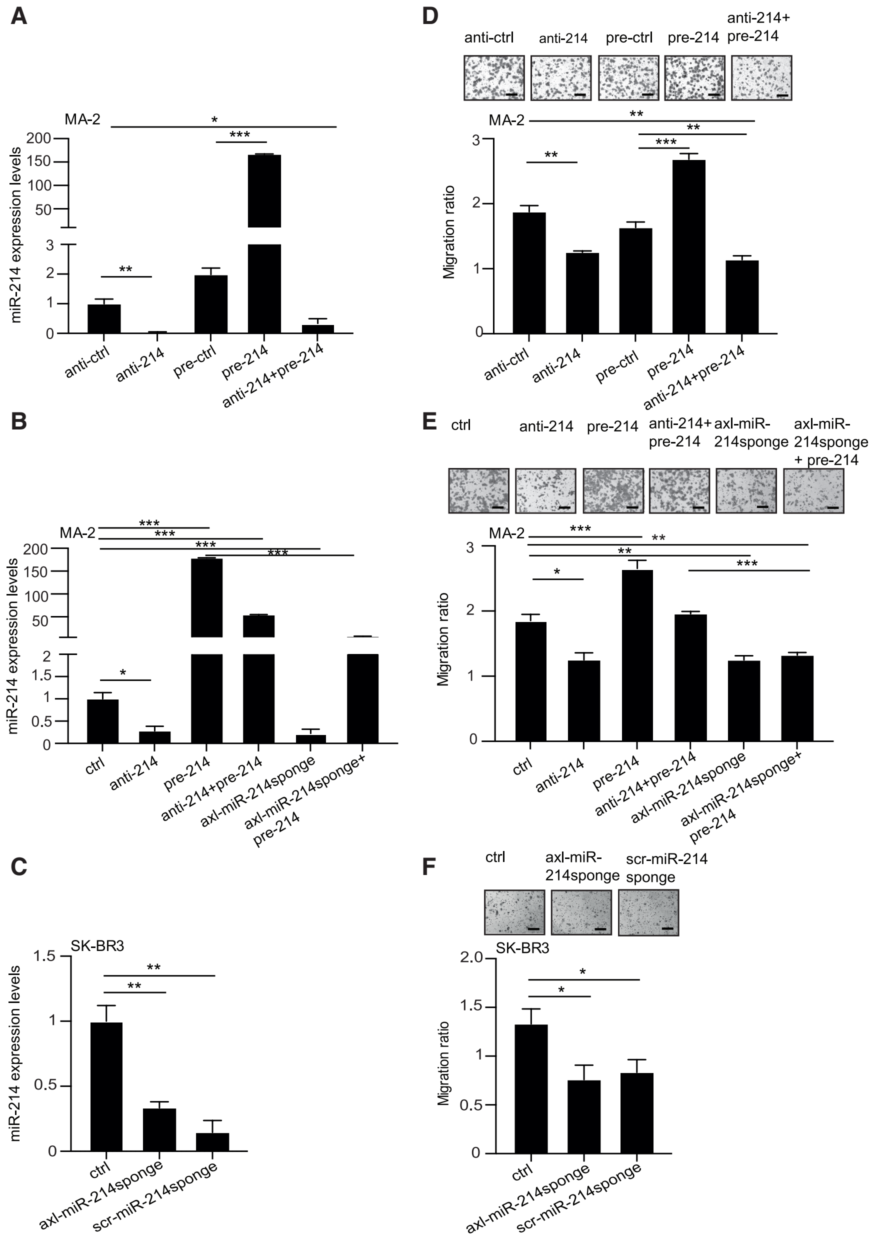

Figure 2. Axl-miR-214sponge chimeric aptamer controls miR-214 levels and cell migration specifically in *axl*-expressing cancer cells

(A–C) miR-214 relative expression levels for *axl*-positive MA-2 cells (A and B) following treatments with control (ctrl) solution, axl aptamer alone, axl-miR-214sponge or scr-miR-214sponge conjugates or for *axl*-negative SK-BR3 cells (C) previously transfected with axl-miR-214sponge or scr-miR-214sponge conjugates or left untreated (ctrl), evaluated by RT-qPCR analysis. Transfection with anti-miR-214 (anti-214) or pre-miR-214 (pre-214) or a combination of the two sequences or their relative controls (anti-ctrl or pre-ctrl) were also performed (A and B) and miR-214 expression levels analyzed by RT-qPCR analysis. Results are shown as fold changes (mean ± SD) relative to controls, normalized on U44 or U6 small nucleolar RNA levels. (D–F) Transwell migration assays for MA-2 (D and E) and *axl*-negative SK-BR3 (F) treated/transfected as described in (A–C). Top: representative photographs of migrated cells. Bottom: graphs of results expressed as ratio of mean ± SEM of the area covered by migrated versus plated cancer cells. At least three independent experiments (in triplicate) were performed, and representative results are shown. *$p < 0.05$; **$p < 0.01$; ***$p < 0.001$; scale bar, 50 μm.

Axl-miR-214sponge chimeric aptamers block *in vivo* breast cancer and melanoma dissemination

To evaluate the therapeutic effect of *axl*-miR-214sponge chimeric aptamers on melanoma or breast cancer dissemination in mice, we injected MA-2 melanoma or 4175-TGL breast cancer cells (tRFP-positive), eventually pretreated for 24 h with axl aptamer or axl-miR-214sponge conjugates subcutaneously or into the mammary gland fat pad for the specified number of NOD/SCID/IL-2R-null (NSG) immunocompromised mice. Then, PBS or axl or axl-miR-214sponge or scr-miR-214sponge solutions were delivered as indicated three times per week, starting at day 2 or 3 post-injection, either into the primary tumor mass or in the blood circulation. To reduce renal filtration of our conjugates, and therefore prolong their persistance in the blood circulation, in some cases a PEGylation (polyethylene glycol) was added to them, as indicated in the figures. Primary tumor growth and number of lung or liver metastases or CTCs were analyzed at the end of the experiments (Figures 4A–4C and S6A–S6D). While tumor growth was similar for all animals analyzed, reduced lung and liver metastasis formation or number of CTCs was observed in mice treated with axl-miR-214sponge compared to the counterparts treated with axl alone or with control solutions (PBS or scr-miR-214sponge).

At the end of the experiments, primary tumor characteristics and compound toxicity were evaluated in treated animals, following *in loco* or systemic injections. Hematoxylin and eosin (H&E) staining of primary masses revealed increased necrosis and immunohistochemistry (IHC) analysis for cleaved caspase-3 showed a slight but significant increase in apoptosis; instead, no alteration in proliferation was detected by stainings for the Ki67 nuclear antigen in axl-miR-214sponge-treated tumors (Figures 5A–5C and S7A–S7C). IHC analyses of *axl* expression showed a strong reduction of *axl* levels in mice treated with axl-miR-214sponge, suggesting internalization of the receptor upon aptamer treatments (Figures 5D and S7D). It is relevant that mouse liver, spleen, and kidney morphology (H&E staining) or weight alterations were not detected following *in loco* (data not shown) or

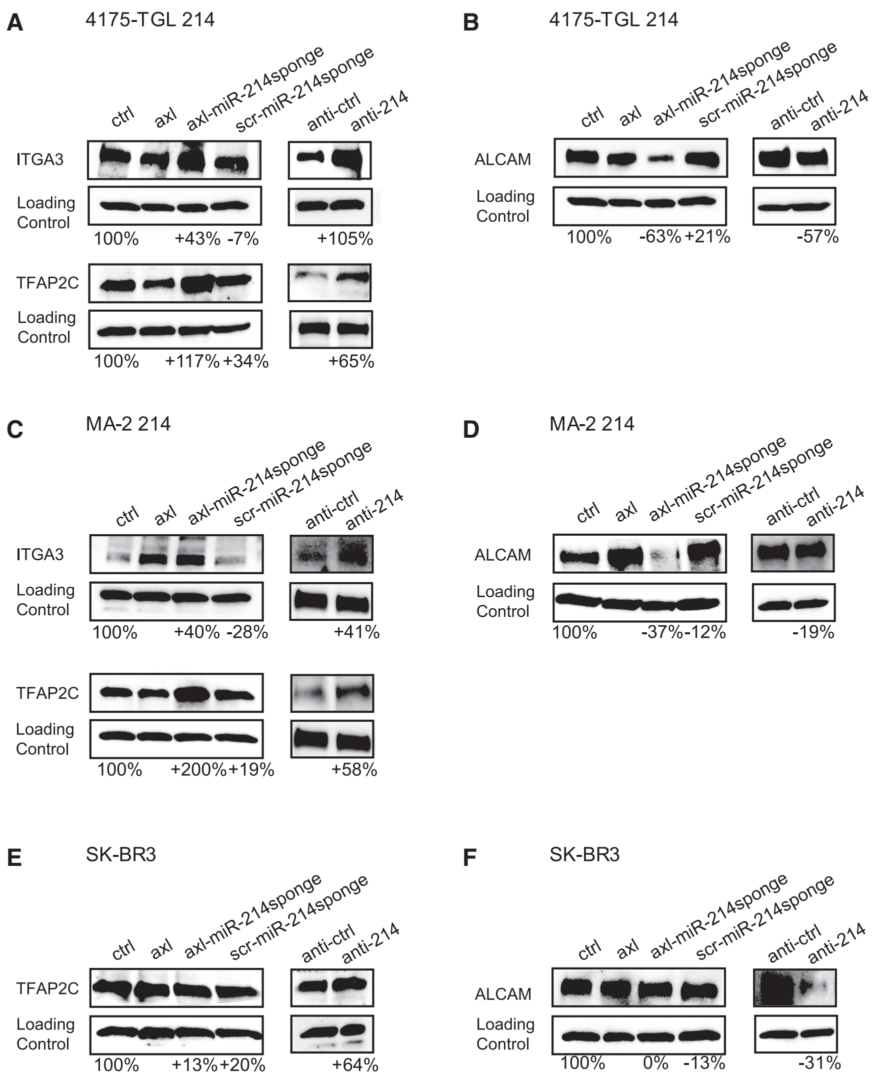

Figure 3. Axl-miR-214sponge conjugate affects miR-214 targets in *axl*-positive breast cancer and melanoma cells

Western blot analysis of miR-214 direct targets ITGA3 and TFAP2C or indirect target ALCAM for *axl*-expressing miR-214 overexpressing 4175-TGL (A and B) or MA-2 (C and D) or *axl*-negative SK-BR3 (E and F) treated with control (ctrl) solution, axl aptamer alone or axl-miR-214sponge or scramble scr-miR-214sponge conjugates for 48 h. Transfection with anti-miR-214 (anti-214) and its relative control (anti-ctrl) was also performed. Protein modulations were calculated relative to axl or anti-control and normalized on loading controls: GAPDH (A, top and bottom left; B–F) or vinculin (A, bottom right) and expressed as percentages. At least three independent experiments were performed, and representative results are shown.

systemic injections, thus excluding toxic effects of the conjugates on mice (Figures 6A–6C and S8A–S8C).

Together, these results suggest that axl-miR-214sponge chimeric conjugates are able to induce cell death in primary tumor masses and to impair cancer cell dissemination while being safe and specific, thus underlying a therapeutic potential for axl-miR-214sponge conjugates in the dissemination control of *axl*-positive cancer cells in the clinic.

DISCUSSION

We previously showed that miR-214 promotes tumor progression in melanoma and breast cancer and dissected its molecular mechanism.[16–18,23] Similarly, other investigators proved the pro-metastatic function of miR-214 in other neoplasia.[24–26] When we investigated the therapeutic potential of miR-214 downmodulation following systemic injections of anti-miR-214 in animals bearing primary breast cancer and melanoma, a strong decrease in metastasis formation and CTCs was seen in mice.[19] The goal of the current work was to develop a tool that could allow delivery of miR-214 inhibitor molecules specifically to breast cancer and melanoma cells. For this purpose, we linked a DNA sequence called *sponge*, capable of trapping and blocking miR-214 activity, to a previously developed and characterized RNA aptamer, GL21.T (axl),[22] able to bind and induce internalization of *axl*, an oncogene tyrosine kinase receptor highly present on the surface of many malignant cancer cells,[27–29] thus generating a chimeric aptamer (or conjugate) named axl-miR-214sponge. The sponge sequence we used was shorter than what was previously tested,[16,18] but nevertheless promising. In fact, by performing *in vitro* and *in vivo* experiments, we proved that axl-miR-214sponge conjugates are able to inhibit metastatic traits of breast cancer and melanoma cells *in vitro*, and, more important, to inhibit metastasis formation and CTCs upon multiple deliveries in mice bearing breast cancer and melanoma primary tumors, with no toxicity for the animals.

Chimeric aptamers were previously reported to be potential therapeutic tools to fight cancer[30,31] or to improve other diseases[32,33] in preclinical studies. Our conjugate is the first example of a composed molecule in which an aptamer is linked to a sponge sequence. Until now, sponge constructs were delivered using viral vectors[34,35] or non-viral systems, including liposomes, artificial polymers, and polysaccharides.[36,37] It is important to emphasize that aptamers are much safer tools than viral particles to deliver molecules[38–40] and that chimeric aptamers composed by DNA/RNA hybrids were previously described as functional.[41,42]

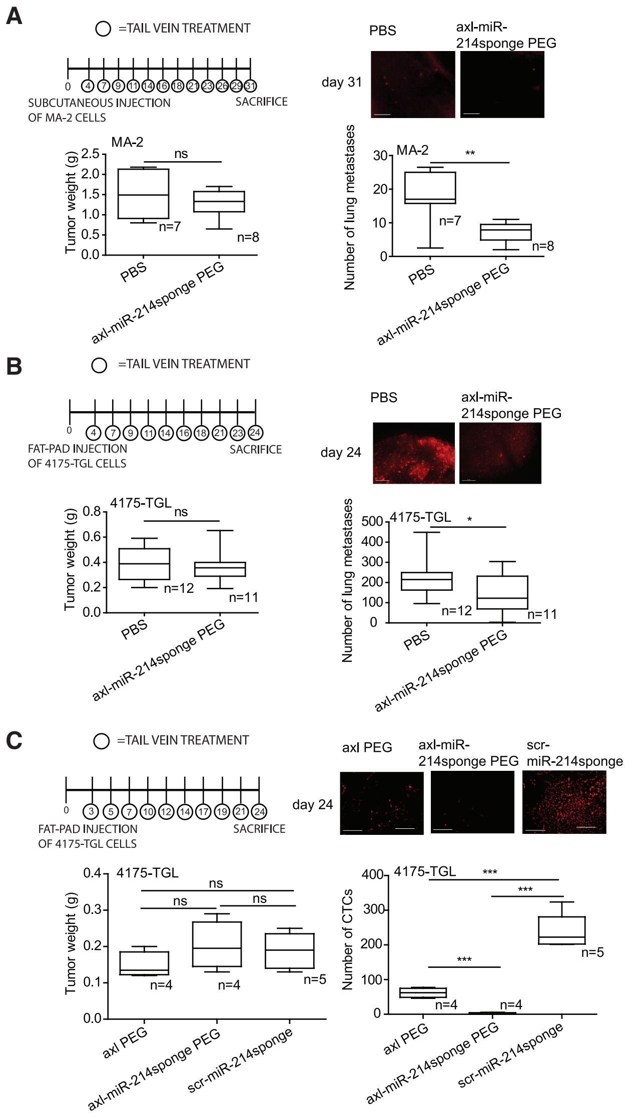

Figure 4. Axl-miR-214sponge PEG conjugate systemic treatments decrease melanoma and breast cancer dissemination in mice

Schemes of the experiments: red fluorescent (RFP-expressing) MA-2 melanoma (A) or 4175-TGL breast cancer (B and C) cells were injected, respectively, subcutaneously or into the mammary gland fat pad in NOD/SCID/IL-2R null mice. Then, PBS or axl or axl-miR-214sponge or scr-miR-214sponge aptamers (with or without PEG) were administered via tail vein injection starting from 3 to 4 days post-injection (3 treatments/week, 1,600 pmol in 100 μL, 10 injections in total). Tumor weight (in grams) and circulating tumor cells (CTCs) or lung metastases were analyzed either 31 or 24 days post-tumor-cell injections and are shown as mean ± SEM for the indicated number of mice. Representative images of fluorescent lung metastases or CTCs are shown. $^{*}p < 0.05$; $^{**}p < 0.01$; $^{***}p < 0.001$; scale bar, 2 mm.

We showed that axl-miR-214sponges inhibit cancer cell migration, invasion, and extravasation (assayed via transendothelial migration assays) *in vitro* similar to other miR-214 inhibitors.[19,43–45] We and others observed that the GL21.T aptamer impairs cell movement as other *axl* inhibitors.[20,22,46,47] However, we showed that when axl and miR-214 were blocked at the same time by using axl-miR-214sponge conjugates, the effect on migration was stronger; instead no effect on cancer cell proliferation was revealed, in line with previous data.[17,18] We believe that the observed synergy between GL21.T and miR-214sponges stems from common functions of *axl* and miR-214 on cancer malignancy and dissemination performed by controlling overlapping but also independent pathways. In fact, *axl* is known to activate migration- and motility-associated pathways, including the epithelial-to-mesenchymal transition, invasion, metastasis formation, and stemness,[48] similar to miR-214.[49] In line with this, axl-miR-214sponge chimeric aptamer administration modulates miR-214 direct targets, such as TFAP2C and ITGA3, but it also acts on other downstream effectors—for instance, on ALCAM, directly targeted by the anti-metastatic miR-148b,[50] as in the introduction.[17,18]

Most important, axl-miR-214sponge chimeric aptamers were able to reduce tumor dissemination of breast cancer or melanoma cells both when administered *in loco* or systemically in mice, and they could also increase necrosis and apoptosis in primary tumors without affecting proliferation (Ki67 staining). Similarly, our previous investigations on cancer cells in culture showed no Ki67 modulation following axl-miR-214sponge treatments.[16] Considering the reduced stability of our chimeric aptamers in presence of serum, often a PEGylation was added to them when delivered to mice to reduce renal filtration and thus prolong their presence in the blood circulation.[51,52] These compounds showed an improved ability to counteract tumor progression compared to non-PEGylated molecules (used in the early experiments, not shown here), thus suggesting the advantage of using modified compounds, also considering the lack of toxicity, as evidenced by the analyses performed on mouse liver, spleen, and kidneys. Additional chemical modifications could be introduced to improve compound stability and thus their efficiency.[21,53]

For all experiments, the *axl*-negative SK-BR3 cells were treated with axl aptamers (axl and axl-miR-214sponge), and no effects were observed. Instead, the effects of miR-214 silencing were present when the same cells were transfected with anti-miR-214, thus indicating aptamer specificity for *axl*-positive cells. axl-miR-214sponge was effective on SK-BR3 only if *axl* expression was imposed. In addition, no effects were detected when an unrelated/scramble chimeric aptamer was used, further reinforcing the selectivity of axl-miR-214sponge conjugate, highly useful for the transfer to the clinics.

Our data demonstrate that the axl aptamer GL21.T present in the axl-miR-214sponge conjugate (with or without PEG) works in a selective manner to convey miR-214sponge inside breast cancer and melanoma cells, thereby reducing off-target effects and damage in healthy tissues. Following delivery in cancer cells, the conjugate is capable of reducing cell metastatic traits and dissemination to distant organs, thus representing a promising tool for breast cancer and melanoma-targeted therapy. In addition to its relevant therapeutic potential for its solo use, our compound could be applied in

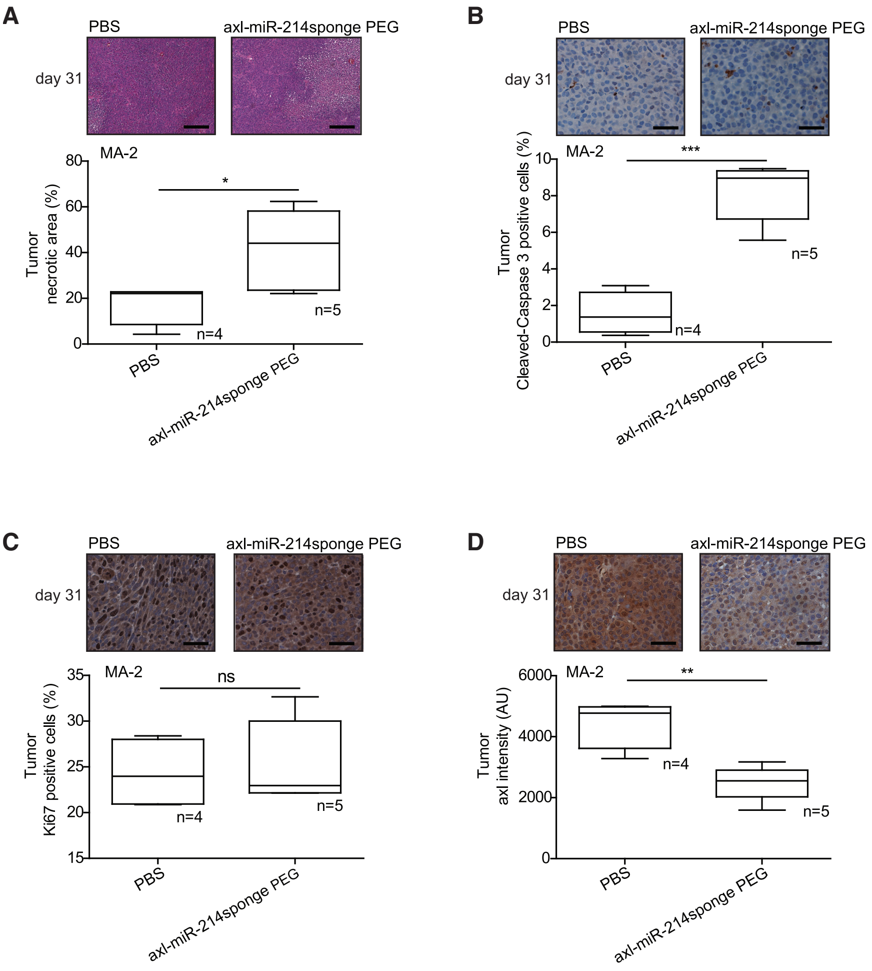

Figure 5. Axl-miR-214sponge chimeric aptamer induces necrosis and apoptosis in melanoma xenotransplants

FFPE sections of MA-2 cell-derived primary tumors treated as in Figure 4A were stained with (A) H&E and necrotic areas evaluated. Representative images are shown on top of plots presenting the percentage of necrotic versus total areas shown as mean ± SEM for the indicated number of mice (10 fields/each mouse). Primary tumors were also stained for (B) cleaved caspase-3, (C) Ki67, or (D) *axl*, and nuclei were counterstained with hematoxylin (blue). Representative pictures are shown on top of plots presenting the percentage of positive versus total cells (A–C) or staining intensity (D) shown as mean ± SEM for the indicated number of mice (10 fields/each mouse). $^{*}p < 0.05$; $^{**}p < 0.01$; $^{***}p < 0.001$; scale bars, 100 μm (A) and 25 μm (B–D).

combination with other therapeutic tools. Because we previously generated an axl-148b conjugate able to inhibit tumor cell spreading by leading to increased miR-148b levels,[20] we could consider using both of our conjugates on the same cells/tumors to further reinforce the impairment of cancer spreading.

MATERIALS AND METHODS

Cell cultures

SK-BR3 came from American Type Culture Collection; 4175-TGLs were kindly provided by J. Massagué[54] and were maintained in standard conditions. SUM-149 cells were a gift of Prof. S. P. Ethier and were cultured as in the paper by Wang et al.[55] L. Xu and R.O. Hynes generously provided us MA-2 cells[56] that were maintained as in the papers by Penna et al.[16,17] WK-MEL was provided by P. Circosta (Molecular Biotechnology Center, Torino, Italy) and maintained in RPMI 1640 medium containing 10 mM GlutaMax and 4.5 g/L glucose (DMEM GlutaMax, GIBCO Invitrogen Life Technologies), supplemented with 10% heat-inactivated fetal bovine serum (FBS) (Servomed GmbH), 25 mM HEPES pH 7.4, and 100 μg/mL gentamicin (all from GIBCO Invitrogen Life Technologies). HUVECs were gently procured by M.F. Brizzi and maintained in M199 medium supplemented with 20% FBS, endothelial cell growth supplement from bovine neural tissue (Sigma), and porcine heparin.

Reagents and antibodies

The reagents and antibodies that were used follow. The anti-miR miRNA inhibitors were anti-miR miRNA Inhibitor Negative Control no. 1 and anti-miR miRNA Inhibitor hsa-miR-214 (AM12124); pre-miR miRNA precursor: pre-miR miRNA Precursor Negative Control no. 1, pre-miR miRNA Precursor Hsa-miR-214 (PM12124) (all from Thermo Fisher); and Axl cDNA expression vector pCMV-Axl (Origene). The primary antibodies were anti-ITGA3, kindly provided by the laboratory of E. Turco (Molecular Biotechnology Center, University of Torino), anti-ITGA5 kindly provided by G. Tarone (Molecular Biotechnology Center, University of Torino), anti-CD166/ALCAM mAb MOG/07 purchased from Novocastra Laboratories, anti-cleaved caspase-3 (Asp175) from Cell Signaling Technologies (catalog no. 9661), anti-Ki67 from Abcam (catalog no. ab 15580), anti-AXL from R&D Systems, and anti-AP2γ (6E4/4), anti-GAPDH pAb V-18, and anti-vinculin (H-300) from Santa Cruz Biotechnology. The secondary antibodies were horseradish peroxidase (HRP)-conjugated goat anti-mouse immunoglobulin G (IgG) and goat anti-rabbit IgG (Santa Cruz Biotechnology).

Chimeric aptamer preparation

To generate axl-miR-214sponge conjugates, a miR-214sponge was linked to the anti-*axl* aptamer GL21.T[22] via sticky-end annealing. Briefly, the RNA GL21.T aptamer (with or without 1 U PEG) and the DNA miR-214sponge were both elongated at their 3′ ends with complementary 17-mer sequences through which they were annealed, forming an RNA/DNA chimeric aptamer.

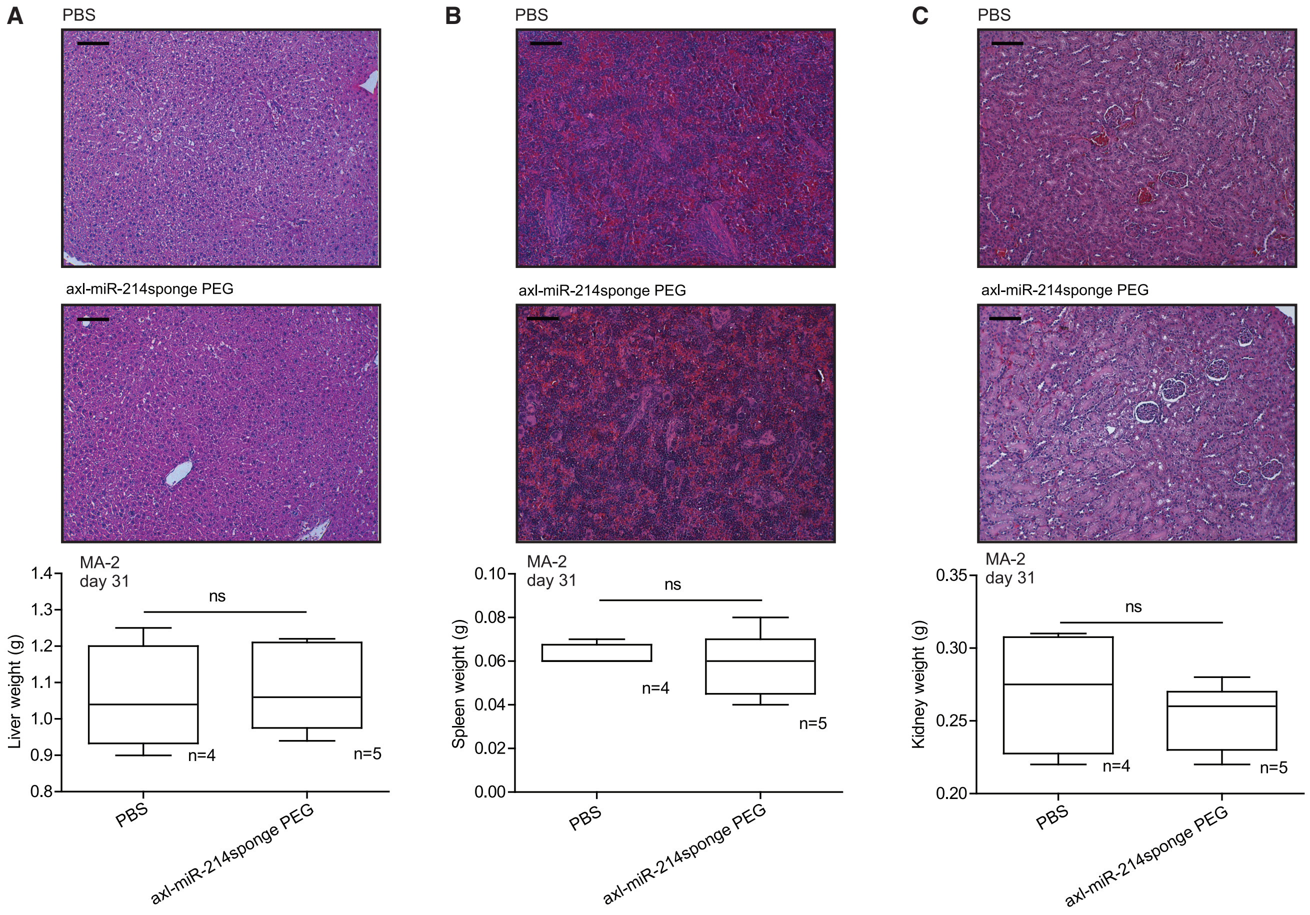

Figure 6. Axl-miR-214sponge PEG conjugate is not toxic for mice when delivered systemically

Liver (A), spleen (B), and kidneys (C) derived from the MA-2 cells experiment described in Figure 4A were weighted at day 31 (final point) and sections stained with H&E. Representative pictures (scale bar, 100 or 200 μm) are shown on top of plots representing the mean ± SEM of organ weights for the indicated number of mice.

A GL21.T scramble sequence was used as negative control. In detail, the sequences used are as follows:

(1) GL21.T-sticky (axl aptamer with sticky sequence underlined): 5′-AUGAUCAAUCGCCUCAAUUCGACAGGAGGCUCACXXXXGUACAUUCUAGAUAGCC-3′
(2) GL21.T-sticky 5′ PEG (a 5′ PEG-axl aptamer with sticky sequence underlined): 5′-PEG-AUGAUCAAUCGCCUCAAUUCGACAGGAGGCUCACXXXXGUACAUUCUAGAUAGCC-3′
(3) GL21.T-scr-sticky (a scramble negative control aptamer, linked to a sticky sequence, as underlined): 5′-GGCGCUAGAACCUUCUAAGCGAAUACAUUACCGCXXXXGUACAUUCUAGAUAGCC-3′
(4) miR-214sponge-sticky (a miR-214sponge linked to a sticky sequence, as underlined): 5′-ACTGCCTGTCCATCCTGCTGTCCTCTTCATCGTCAAACTGCCTGTCAAACCTGCTGTAATACCCGGCTATCTAGAATGTAC- 3′

The sponge used here corresponds to a shorter version of a previously designed and successfully used miR-214sponge sequence composed of eight miR-214 binding sites (21 nt each) interrupted by 15 nucleotide spacers, perfectly complementary to the miR-214 seed region, with a bulge in position 9–12.[16,18] The sequence was shortened to reduce steric hindrance and preserve the folding of the complex. To improve stability, all RNAs were modified with 2′-fluoro pyrimidines (2′-F-Py). All sequences were generated at the Synthetic and Biopolymer Chemistry Core, Beckman Research Institute City of Hope (Duarte, CA). Sticky sequences are underlined and contain 2′-F-Py and 2′-*O*-methylpurine 2′. “X” indicates the C3 carbon linkers. In an alternative formulation, a 20-kDa PEG molecule was added at the 5′ end of the GL21.T-sticky molecule. All chimeric aptamers were prepared as follows: (1) miR-214sponge was incubated at 95°C for 10 min; (2) axl or axl-PEG or scramble aptamers were refolded (5 min at 85°C, 2 min on ice, 10 min at room temperature); and (3) equal amounts of aptamers and miR-214sponge were annealed by incubating

Table 1. TaqMan primer assays used in the study

Assay	ID	Catalog no.
miR-214	002306	4427975
RNU44	001094	4427975
U6 snRNA	001973	4427975

them together at 37°C for 30 min. The annealing efficiency was controlled using 12% non-denaturing PAGE for each preparation.

Transient transfections, stable cell lines, and treatments

To obtain transient miRNA or *axl* expression or aptamer conjugates, cells were plated at 80% confluency and transfected 24 h later with Lipofectamine 2000 and 75 nmol/L anti-miRNA or pre-miRNA (Invitrogen Life Technologies) or 1 μg pCMVaxl (Origene) expression vector or pCMV-empty vector or 400 nmol/L of folded axl, axl-miR-214sponge, or scr-miR-214sponge aptamers. All stable cell lines were generated via lentiviral infections according to Trono's laboratory protocol (École Polytechnique Fédérale de Lausanne, Lausanne, Switzerland, http://tronolab.pfl.ch). For aptamer alone or conjugate administration, cells were plated in 24-well dishes at 80% confluency and treated 24 h later with 400 nmol/L of the folded axl, axl-miR-214sponge, or scr-miR-214sponge aptamers or treated with the aptamer binding buffer solution (ctrl). For SK-BR3 co-treatment experiments, aptamer alone or conjugates were added 6 h after pCMVAxl and pCMV-empty vector transfection.

Axl-miR-214sponge chimeric aptamer stability in human serum

To investigate the chimera stability, the conjugate was incubated in human serum (Type AB Human Serum, Euroclone catalog no. ECS0219D) from 0 to 168 h. At each time point (0, 1, 2, 4, 8, 24, 48, 72, and 168 h), 8 μL 80% serum solution (32 pmol) was collected, 1 μL Proteinase K solution (600 mAU/mL) was added, and the solution was incubated for 1 h at 37°C. Then, 9 μL 1× Tris/Borate/EDTA (TBE) and 3 μL loading buffer (Invitrogen) were added to the samples and stored at −80°C. Finally, the collected samples were separated by electrophoresis into 12% non-denaturing PAG stained with ethidium bromide.

Isolation of proteins or RNA, WB, qRT-PCRs

Boiling buffer containing 0.125 M Tris/HCl, pH 6.8, and 2.5% sodium dodecyl sulfate (SDS) was used to extract total proteins. For WB, 20–50 μg proteins were separated by SDS-PAGE and electroblotted onto nitrocellulose membranes (BioRad). Then, the membranes were blocked with 5% non-fat milk PBS-Tween 0.1% buffer (4.3 mM sodium phosphate, dibasic Na_2HPO_4, 137 mM sodium chloride, 2.7 mM potassium chloride, 1.4 mM potassium phosphate, monobasic KH_2PO_4, pH 7.4, with 0.1% Tween 20) for 1 h at room temperature and incubated with primary (overnight at 4°C) and secondary antibodies (1 h at room temperature) in PBS-Tween 0.1% buffer, and visualized by enhanced chemiluminescence (GE Healthcare) using the Chemidoc Touch Imaging System (Bio-Rad). TRIzol reagent (Invitrogen Life Technologies) was used to isolate total RNA. RT-qPCRs were performed to detect miRNAs with the indicated TaqMan MicroRNA Assays (Applied Biosystems) on 10 ng RNA. Quantitative normalization was performed on RNU44 or U6 small nucleolar RNA. The relative expression levels between samples were calculated using the delta Ct (threshold cycle number) method (2-ΔΔCt) with a control sample as the reference point.[57] The TaqMan primer assays used in the study are listed in Table 1.

Proliferation, viability migration, invasion, and transendothelial migration assays

In vitro proliferation, migration, invasion, and transendothelial migration assays were performed as in the studies of Penna et al. and Cerchia et al.[16,17,22]

In vivo tumor growth and metastasis assays

All experiments performed with live animals complied with ethical care. The number of the project approved by the Italian Health Ministry is CC652.140. NSG mice were injected with 5×10^6 4175-TGL breast cancer melanoma cells pre-treated the day before the injection with 400 nM of axl aptamer alone or axl-miR-214sponge into the mammary fat pad. Two days after the injection, tumors were treated with axl or axl-miR-214sponge (300 pmol/injection, three injections per week). Mice were sacrificed and dissected 23 days after cell injections. Alternatively, NSG mice were injected with either 5×10^6 4175-TGL breast cancer or MA-2 melanoma cells, respectively, into the mammary fat pad or subcutaneously. Then, mice were treated with PBS or axl or axl-miR-214sponge or scr-miR-214sponge, with or without PEG (1,600 pmol/injection, three injections per week) starting from days 3 or 4 after tumor cell injection. Mice were sacrificed and dissected 24 or 31 days after cell injections. For both assays, primary tumor weight/morphology, CTCs, and lung or liver metastases were evaluated. Liver, spleen, and kidney weights and morphology were analyzed at the endpoint.

Histology and IHC

Tumors were dissected and formalin fixed and paraffin embedded (FFPE). Then, 5-μm-thick tissue sections were generated and stained with H&E for histology observations. IHC stainings were performed using anti-Ki67, anti-cleaved caspase-3, or anti-*axl* antibodies and revealed with avidin-biotin-peroxidase techniques (Anti-Mouse HRP-DAB Cell & Tissue Staining Kit, R&D Systems). All slides were counterstained with hematoxylin.

Isolation of CTCs

For the isolation of CTCs, blood was collected from heart-punctured mice and kept in culture with normal medium for 3 days. Then, attached cells were washed with PBS and cultured in medium with puromycin to select antibiotic-resistant tumor cells. After 7 days, cells were washed, and endogenous fluorescence was evaluated with a fluorescent microscope.

TCGA dataset mRNA analysis

TCGA data for melanoma (SKCM) and breast cancer (BRCA) patients were downloaded through the TCGAbiolinks package with the following settings: data.category = “Transcriptome Profiling,” data.type = “Gene Expression Quantification,” workflow.type = “STAR – Counts,” and experimental.strategy = “RNA-Seq” assay “fpkm_uq_unstrand.” Gene IDs were mapped to gene symbols, and in the case of multiple IDs matching the same gene symbol, the ID with the highest average expression was kept. Melanoma samples were divided by sample type (primary, metastatic), and samples of the same type belonging to the same patient were averaged. Data were log transformed with an offset of 1. For breast cancer samples, clinical data and molecular subtypes were obtained with the GDCquery_clinic and TCGAquery_subtype functions, respectively. Plots were generated with ggplot2.

Statistical analysis

Results are presented as mean ± standard deviation (SD) or mean ± standard error of the mean (SEM), and a two-tailed Student’s t test was used for comparisons. $*p < 0.05$,$**p < 0.01$, and $***p < 0.001$ were considered statistically significant, and ns indicates a p value that is not statistically significant.

DATA AND CODE AVAILABILITY

The data generated and analyzed during this study are available upon reasonable request to the corresponding authors.

ACKNOWLEDGMENTS

The research leading to these results has received funding from AIRC under IG 2022-ID 27054 project, principal investigator (PI): D.T.; the National Center for Gene Therapy and Drugs based on RNA Technology, Spoke2, PNRR M4C2-Investimento 1.4-CN00000041 to D.T.; PoC Instrument 2020 to D.T.; PoC TOINPROVE/2023 to D.T.; Ex-POST 21 San SanPaolo/UNITO to D.T.; Italian Ministry of Health (RF-2016-02361048 to D.T.); DEFLeCT, Regione Piemonte 320-44 to D.T.; IPA Award 2021 to D.T.; Fondazione CRT Torino 2018.1311 to D.T.; Progetto di Ricerca di Ateneo 2017/SanPaolo Torino (CST0165134 to D.T.); RILO Unito to D.T. 2021, 2022, 2023, 2024 (ex−60%); Fondazione CRT Torino-CUPE19C20001260007 to F.O.; Italian University and Research Ministry (MUR) program “Departments of Excellence 2023–2027” to F.O.; AGING Project 2023–Department of Translational Medicine, University of Piemonte Orientale to F.O.; AIRC IG – ID 28703 (PI: C.L.E.); AIRC IG – ID 29052 (PI: S.C.); Italian Ministry of University and Research 2022BFF2CJ (to C.L.E.); WorldWide Cancer Research 22-0129 (to C.L.E.); MUR-PRIN 2022 project number 2022WYAEWE to P.D.; AIRC under IG 2022 - ID 27353 project (PI: P.D.); Italian Ministry of Health (RF-2021-12371961) to P.D.; and PNRR M4C2-Investimento 1.4-CN00000041 “Finanziato dall’Unione Europea-NextGenerationEU” to P.D. L.Q. was a recipient of a 3-year FIRC-AIRC fellowship for Italy (ID 24188) and of two short-term travel fellowships offered by EACR and SIBBM. P.D.R.C. was a recipient of a Veronesi fellowship. S.R. is an RTDA (fixed-term researcher of type A) supported by the above-mentioned PNRR M4C2-Investimento 1.4-CN00000041. We are grateful to L. Xu and R.O. Hynes for the MA-2 cells; J. Massagué for the 4175-TGL cells; M.F. Brizzi for the HUVECs; S.P. Ethier for the SUM-149 cells; P. Circosta for the WK-MEL cells; G. Tarone (deceased) for the anti-ITGA5 pAb RM10; E. Turco for the anti-ITGA3 antibody; and F. Cristofani for his help with the immunocompromised mice.

AUTHOR CONTRIBUTIONS

Conceptualization, D.T., L.Q., and F.O.; methodology, L.Q., F.O., and S.R.; investigation, L.Q., F.O., N.V., S.B., P.D.R.C., A.S., S.C., and S.R.; writing, reviewing, and editing, D.T., L.Q., S.R., and F.O.; figure preparation and data analysis, L.Q., F.O., and S.R.; supervision, D.T., C.L.E., V.d.F., P.D., and F.O.; project administration, D.T.; and sponge design, M.B.S. All authors have read and agreed to the published version of the manuscript.

DECLARATION OF INTERESTS

The chimeric aptamer axl-miR-214sponge has been patented (IT102021000022610).

SUPPLEMENTAL INFORMATION

Supplemental information can be found online at https://doi.org/10.1016/j.ymthe.2025.07.039.

REFERENCES

1. Lei, Z.N., Tian, Q., Teng, Q.X., Wurpel, J.N.D., Zeng, L., Pan, Y., and Chen, Z.S. (2023). Understanding and targeting resistance mechanisms in cancer. MedComm *4*, e265. https://doi.org/10.1002/mco2.265.
2. He, Y., Zheng, J., Ye, B., Dai, Y., and Nie, K. (2023). Chemotherapy-induced gastrointestinal toxicity: Pathogenesis and current management. Biochem. Pharmacol. *216*, 115787. https://doi.org/10.1016/j.bcp.2023.115787.
3. Lustberg, M.B., Kuderer, N.M., Desai, A., Bergerot, C., and Lyman, G.H. (2023). Mitigating long-term and delayed adverse events associated with cancer treatment: implications for survivorship. Nat. Rev. Clin. Oncol. *20*, 527–542. https://doi.org/10.1038/s41571-023-00776-9.
4. Siaravas, K.C., Katsouras, C.S., and Sioka, C. (2023). Radiation Treatment Mechanisms of Cardiotoxicity: A Systematic Review. Int. J. Mol. Sci. *24*, 6272. https://doi.org/10.3390/ijms24076272.
5. Liang, X.W., Liu, B., Chen, J.C., Cao, Z., Chu, F.R., Lin, X., Wang, S.Z., and Wu, J.C. (2023). Characteristics and molecular mechanism of drug-tolerant cells in cancer: a review. Front. Oncol. *13*, 1177466. https://doi.org/10.3389/fonc.2023.1177466.
6. Kinnel, B., Singh, S.K., Oprea-Ilies, G., and Singh, R. (2023). Targeted Therapy and Mechanisms of Drug Resistance in Breast Cancer. Cancers (Basel) *15*, 1320. https://doi.org/10.3390/cancers15041320.
7. Said, S.S., and Ibrahim, W.N. (2023). Cancer Resistance to Immunotherapy: Comprehensive Insights with Future Perspectives. Pharmaceutics *15*, 1143. https://doi.org/10.3390/pharmaceutics15041143.
8. Bartel, D.P. (2009). MicroRNAs: target recognition and regulatory functions. Cell *136*, 215–233. https://doi.org/10.1016/j.cell.2009.01.002.
9. Zhang, X., Xu, X., Song, J., Xu, Y., Qian, H., Jin, J., and Liang, Z.F. (2023). Non-coding RNAs’ function in cancer development, diagnosis and therapy. Biomed. Pharmacother. *167*, 115527. https://doi.org/10.1016/j.biopha.2023.115527.
10. Doghish, A.S., Elazazy, O., Mohamed, H.H., Mansour, R.M., Ghanem, A., Faraag, A. H.I., Elballal, M.S., Elrebehy, M.A., Elesawy, A.E., Abdel Mageed, S.S., et al. (2023). The role of miRNAs in multiple sclerosis pathogenesis, diagnosis, and therapeutic resistance. Pathol. Res. Pract. *251*, 154880. https://doi.org/10.1016/j.prp.2023.154880.
11. Choi, D., Kim, J., Yang, J.W., Kim, J.H., Park, S., and Shin, J.I. (2023). Dysregulated MicroRNAs in the Pathogenesis of Systemic Lupus Erythematosus: A Comprehensive Review. Int. J. Biol. Sci. *19*, 2495–2514. https://doi.org/10.7150/ijbs.74315.
12. Li, S., Lei, Z., and Sun, T. (2023). The role of microRNAs in neurodegenerative diseases: a review. Cell Biol. Toxicol. *39*, 53–83. https://doi.org/10.1007/s10565-022-09761-x.
13. Santovito, D., Fan, Y., Elia, L., Tan, J.T.M., and van der Vorst, E.P.C. (2023). Editorial: Emerging roles of miRNAs in cardiovascular disease. Front. Cardiovasc. Med. *10*, 1144849. https://doi.org/10.3389/fcvm.2023.1144849.
14. Kim, T., and Croce, C.M. (2023). MicroRNA: trends in clinical trials of cancer diagnosis and therapy strategies. Exp. Mol. Med. *55*, 1314–1321. https://doi.org/10.1038/s12276-023-01050-9.
15. Quirico, L., and Orso, F. (2020). The power of microRNAs as diagnostic and prognostic biomarkers in liquid biopsies. Cancer Drug Resist. *3*, 117–139. https://doi.org/10.20517/cdr.2019.103.
16. Penna, E., Orso, F., Cimino, D., Tenaglia, E., Lembo, A., Quaglino, E., Poliseno, L., Haimovic, A., Osella-Abate, S., De Pittà, C., et al. (2011). microRNA-214 contributes to melanoma tumour progression through suppression of TFAP2C. EMBO J. *30*, 1990–2007. https://doi.org/10.1038/emboj.2011.102.
17. Penna, E., Orso, F., Cimino, D., Vercellino, I., Grassi, E., Quaglino, E., Turco, E., and Taverna, D. (2013). miR-214 coordinates melanoma progression by upregulating

ALCAM through TFAP2 and miR-148b downmodulation. Cancer Res. *73*, 4098–4111. https://doi.org/10.1158/0008-5472.CAN-12-3686.

18. Orso, F., Quirico, L., Virga, F., Penna, E., Dettori, D., Cimino, D., Coppo, R., Grassi, E., Elia, A.R., Brusa, D., et al. (2016). miR-214 and miR-148b Targeting Inhibits Dissemination of Melanoma and Breast Cancer. Cancer Res. *76*, 5151–5162. https://doi.org/10.1158/0008-5472.CAN-15-1322.
19. Dettori, D., Orso, F., Penna, E., Baruffaldi, D., Brundu, S., Maione, F., Turco, E., Giraudo, E., and Taverna, D. (2018). Therapeutic Silencing of miR-214 Inhibits Tumor Progression in Multiple Mouse Models. Mol. Ther. *26*, 2008–2018. https://doi.org/10.1016/j.ymthe.2018.05.020.
20. Quirico, L., Orso, F., Esposito, C.L., Bertone, S., Coppo, R., Conti, L., Catuogno, S., Cavallo, F., de Franciscis, V., and Taverna, D. (2020). Axl-148b chimeric aptamers inhibit breast cancer and melanoma progression. Int. J. Biol. Sci. *16*, 1238–1251. https://doi.org/10.7150/ijbs.39768.
21. Amero, P., Lokesh, G.L.R., Chaudhari, R.R., Cardenas-Zuniga, R., Schubert, T., Attia, Y.M., Montalvo-Gonzalez, E., Elsayed, A.M., Ivan, C., Wang, Z., et al. (2021). Conversion of RNA Aptamer into Modified DNA Aptamers Provides for Prolonged Stability and Enhanced Antitumor Activity. J. Am. Chem. Soc. *143*, 7655–7670. https://doi.org/10.1021/jacs.9b10460.
22. Cerchia, L., Esposito, C.L., Camorani, S., Rienzo, A., Stasio, L., Insabato, L., Affuso, A., and de Franciscis, V. (2012). Targeting Axl with an high-affinity inhibitory aptamer. Mol. Ther. *20*, 2291–2303. https://doi.org/10.1038/mt.2012.163.
23. Orso, F., Virga, F., Dettori, D., Dalmasso, A., Paradzik, M., Savino, A., Pomatto, M. A.C., Quirico, L., Cucinelli, S., Coco, M., et al. (2023). Stroma-derived miR-214 coordinates tumor dissemination. J. Exp. Clin. Cancer Res. *42*, 20. https://doi.org/10.1186/s13046-022-02553-5.
24. Long, H., Wang, Z., Chen, J., Xiang, T., Li, Q., Diao, X., and Zhu, B. (2015). microRNA-214 promotes epithelial-mesenchymal transition and metastasis in lung adenocarcinoma by targeting the suppressor-of-fused protein (Sufu). Oncotarget *6*, 38705–38718. https://doi.org/10.18632/oncotarget.5478.
25. Andreucci, E., Ruzzolini, J., Bianchini, F., Versienti, G., Biagioni, A., Lulli, M., Guasti, D., Nardini, P., Serratì, S., Margheri, F., et al. (2022). miR-214-Enriched Extracellular Vesicles Released by Acid-Adapted Melanoma Cells Promote Inflammatory Macrophage-Dependent Tumor Trans-Endothelial Migration. Cancers (Basel) *14*, 5090. https://doi.org/10.3390/cancers14205090.
26. Amin, M.M.J., Trevelyan, C.J., and Turner, N.A. (2021). MicroRNA-214 in Health and Disease. Cells *10*, 3274. https://doi.org/10.3390/cells10123274.
27. Li, Y., Ye, X., Tan, C., Hongo, J.A., Zha, J., Liu, J., Kallop, D., Ludlam, M.J.C., and Pei, L. (2009). Axl as a potential therapeutic target in cancer: role of Axl in tumor growth, metastasis and angiogenesis. Oncogene *28*, 3442–3455. https://doi.org/10.1038/onc.2009.212.
28. Sensi, M., Catani, M., Castellano, G., Nicolini, G., Alciato, F., Tragni, G., De Santis, G., Bersani, I., Avanzi, G., Tomassetti, A., et al. (2011). Human cutaneous melanomas lacking MITF and melanocyte differentiation antigens express a functional Axl receptor kinase. J. Invest. Dermatol. *131*, 2448–2457. https://doi.org/10.1038/jid.2011.218.
29. Rankin, E.B., and Giaccia, A.J. (2016). The Receptor Tyrosine Kinase AXL in Cancer Progression. Cancers (Basel) *8*, 103. https://doi.org/10.3390/cancers8110103.
30. Dai, F., Zhang, Y., Zhu, X., Shan, N., and Chen, Y. (2013). The anti-chemoresistant effect and mechanism of MUC1 aptamer-miR-29b chimera in ovarian cancer. Gynecol. Oncol. *131*, 451–459. https://doi.org/10.1016/j.ygyno.2013.07.112.
31. Meraviglia-Crivelli, D., Villanueva, H., Menon, A.P., Zheleva, A., Moreno, B., Villalba-Esparza, M., and Pastor, F. (2022). A pan-tumor-siRNA aptamer chimera to block nonsense-mediated mRNA decay inflames and suppresses tumor progression. Mol. Ther. Nucleic Acids *29*, 413–425. https://doi.org/10.1016/j.omtn.2022.07.017.
32. Zhu, Q., Shibata, T., Kabashima, T., and Kai, M. (2012). Inhibition of HIV-1 protease expression in T cells owing to DNA aptamer-mediated specific delivery of siRNA. Eur. J. Med. Chem. *56*, 396–399. https://doi.org/10.1016/j.ejmech.2012.07.045.
33. Wang, C.W., Chung, W.H., Cheng, Y.F., Ying, N.W., Peck, K., Chen, Y.T., and Hung, S.I. (2013). A new nucleic acid-based agent inhibits cytotoxic T lymphocyte-mediated immune disorders. J. Allergy Clin. Immunol. *132*, 713–722.e11. https://doi.org/10.1016/j.jaci.2013.04.036.
34. Wang, X.W., Zhang, C., Lee, K.C., He, X.J., Lu, Z.Q., Huang, C., and Wu, Q.C. (2017). Adenovirus-Mediated Gene Transfer of microRNA-21 Sponge Inhibits Neointimal Hyperplasia in Rat Vein Grafts. Int. J. Biol. Sci. *13*, 1309–1319. https://doi.org/10.7150/ijbs.20254.
35. Wu, K., Ye, C., Lin, L., Chu, Y., Ji, M., Dai, W., Zeng, X., and Lin, Y. (2016). Inhibiting miR-21 attenuates experimental hepatic fibrosis by suppressing both the ERK1 pathway in HSC and hepatocyte EMT. Clin. Sci. *130*, 1469–1480. https://doi.org/10.1042/CS20160334.
36. Tay, F.C., Lim, J.K., Zhu, H., Hin, L.C., and Wang, S. (2015). Using artificial microRNA sponges to achieve microRNA loss-of-function in cancer cells. Adv. Drug Deliv. Rev. *81*, 117–127. https://doi.org/10.1016/j.addr.2014.05.010.
37. Kent, O.A., Steenbergen, C., and Das, S. (2018). In Vivo Nanovector Delivery of a Heart-specific MicroRNA-sponge. J. Vis. Exp. *136*, 57845. https://doi.org/10.3791/57845.
38. Catuogno, S., Esposito, C.L., and de Franciscis, V. (2016). Aptamer-Mediated Targeted Delivery of Therapeutics: An Update. Pharmaceuticals (Basel) *9*, 69. https://doi.org/10.3390/ph9040069.
39. Nimjee, S.M., White, R.R., Becker, R.C., and Sullenger, B.A. (2017). Aptamers as Therapeutics. Annu. Rev. Pharmacol. Toxicol. *57*, 61–79. https://doi.org/10.1146/annurev-pharmtox-010716-104558.
40. Kovacevic, K.D., Gilbert, J.C., and Jilma, B. (2018). Pharmacokinetics, pharmacodynamics and safety of aptamers. Adv. Drug Deliv. Rev. *134*, 36–50. https://doi.org/10.1016/j.addr.2018.10.008.
41. Dai, F., Zhang, Y., Zhu, X., Shan, N., and Chen, Y. (2012). Anticancer role of MUC1 aptamer-miR-29b chimera in epithelial ovarian carcinoma cells through regulation of PTEN methylation. Target. Oncol. *7*, 217–225. https://doi.org/10.1007/s11523-012-0236-7.
42. Tanno, T., Zhang, P., Lazarski, C.A., Liu, Y., and Zheng, P. (2017). An aptamer-based targeted delivery of miR-26a protects mice against chemotherapy toxicity while suppressing tumor growth. Blood Adv. *1*, 1107–1119. https://doi.org/10.1182/bloodadvances.2017004705.
43. Tao, Y., Zhao, Z., Ma, J., Dong, L., Liang, Y., Li, S., Mao, Y., Li, Y., and Zhang, Y. (2019). MiR-214-3p regulates the viability, invasion, migration and EMT of TNBC cells by targeting ST6GAL1. Cytotechnology *71*, 1155–1165. https://doi.org/10.1007/s10616-019-00352-z.
44. Zhang, Y., Zhao, Z., Li, S., Dong, L., Li, Y., Mao, Y., Liang, Y., Tao, Y., and Ma, J. (2019). Inhibition of miR-214 attenuates the migration and invasion of triple-negative breast cancer cells. Mol. Med. Rep. *19*, 4035–4042. https://doi.org/10.3892/mmr.2019.10112.
45. Wang, S.J., Li, W.W., Wen, C.J., Diao, Y.L., and Zhao, T.L. (2020). MicroRNA-214 promotes the EMT process in melanoma by downregulating CADM1 expression. Mol. Med. Rep. *22*, 3795–3803. https://doi.org/10.3892/mmr.2020.11446.
46. Shen, Y., Zhang, W., Liu, J., He, J., Cao, R., Chen, X., Peng, X., Xu, H., Zhao, Q., Zhong, J., et al. (2019). Therapeutic activity of DCC-2036, a novel tyrosine kinase inhibitor, against triple-negative breast cancer patient-derived xenografts by targeting AXL/MET. Int. J. Cancer *144*, 651–664. https://doi.org/10.1002/ijc.31915.
47. Szabadkai, I., Torka, R., Garamvölgyi, R., Baska, F., Gyulavári, P., Boros, S., Illyés, E., Choidas, A., Ullrich, A., and Őrfi, L. (2018). Discovery of N-[4-(Quinolin-4-yloxy)phenyl]benzenesulfonamides as Novel AXL Kinase Inhibitors. J. Med. Chem. *61*, 6277–6292. https://doi.org/10.1021/acs.jmedchem.8b00672.
48. Yadav, M., Sharma, A., Patne, K., Tabasum, S., Suryavanshi, J., Rawat, L., Machaalani, M., Eid, M., Singh, R.P., Choueiri, T.K., et al. (2025). AXL signaling in cancer: from molecular insights to targeted therapies. Signal Transduct. Target. Ther. *10*, 37. https://doi.org/10.1038/s41392-024-02121-7.
49. Penna, E., Orso, F., and Taverna, D. (2015). miR-214 as a key hub that controls cancer networks: small player, multiple functions. J. Invest. Dermatol. *135*, 960–969. https://doi.org/10.1038/jid.2014.479.
50. Cimino, D., De Pittà, C., Orso, F., Zampini, M., Casara, S., Penna, E., Quaglino, E., Forni, M., Damasco, C., Pinatel, E., et al. (2013). miR148b is a major coordinator of breast cancer progression in a relapse-associated microRNA signature by targeting ITGA5, ROCK1, PIK3CA, NRAS, and CSF1. FASEB J. *27*, 1223–1235. https://doi.org/10.1096/fj.12-214692.

51. Veronese, F.M., and Pasut, G. (2008). PEGylation: Posttranslational bioengineering of protein biotherapeutics. Drug Discov. Today Technol. *5*, e57–e64. https://doi.org/10.1016/j.ddtec.2009.02.002.

52. Harris, J.M., Martin, N.E., and Modi, M. (2001). Pegylation: a novel process for modifying pharmacokinetics. Clin. Pharmacokinet. *40*, 539–551. https://doi.org/10.2165/00003088-200140070-00005.

53. Szymanowski, W., Szymanowska, A., Bielawska, A., Lopez-Berestein, G., Rodriguez-Aguayo, C., and Amero, P. (2023). Aptamers as Potential Therapeutic Tools for Ovarian Cancer: Advancements and Challenges. Cancers (Basel) *15*, 5300. https://doi.org/10.3390/cancers15215300.

54. Minn, A.J., Gupta, G.P., Siegel, P.M., Bos, P.D., Shu, W., Giri, D.D., Viale, A., Olshen, A.B., Gerald, W.L., and Massagué, J. (2005). Genes that mediate breast cancer metastasis to lung. Nature *436*, 518–524. https://doi.org/10.1038/nature03799.

55. Wang, Y., Liu, X., Chen, L., Cheng, D., Rusckowski, M., and Hnatowich, D.J. (2009). Tumor delivery of antisense oligomer using trastuzumab within a streptavidin nanoparticle. Eur. J. Nucl. Med. Mol. Imaging *36*, 1977–1986. https://doi.org/10.1007/s00259-009-1201-2.

56. Xu, L., Shen, S.S., Hoshida, Y., Subramanian, A., Ross, K., Brunet, J.P., Wagner, S.N., Ramaswamy, S., Mesirov, J.P., and Hynes, R.O. (2008). Gene expression changes in an animal melanoma model correlate with aggressiveness of human melanoma metastases. Mol. Cancer Res. *6*, 760–769. https://doi.org/10.1158/1541-7786.MCR-07-0344.

57. Fleige, S., Walf, V., Huch, S., Prgomet, C., Sehm, J., and Pfaffl, M.W. (2006). Comparison of relative mRNA quantification models and the impact of RNA integrity in quantitative real-time RT-PCR. Biotechnol. Lett. *28*, 1601–1613. https://doi.org/10.1007/s10529-006-9127-2.

YMTHE, Volume *33*

Supplemental Information

The chimeric aptamer axl-miR-214sponge inhibits breast cancer and melanoma dissemination

Lorena Quirico, Sabrina Rizzolio, Sofia Bertone, Priscila D.R. Cirillo, Aurora Savino, Nicoletta Vitale, Silvia Catuogno, Carla L. Esposito, Michael B. Stadler, Paola Defilippi, Vittorio de Franciscis, Francesca Orso, and Daniela Taverna

Figure S1

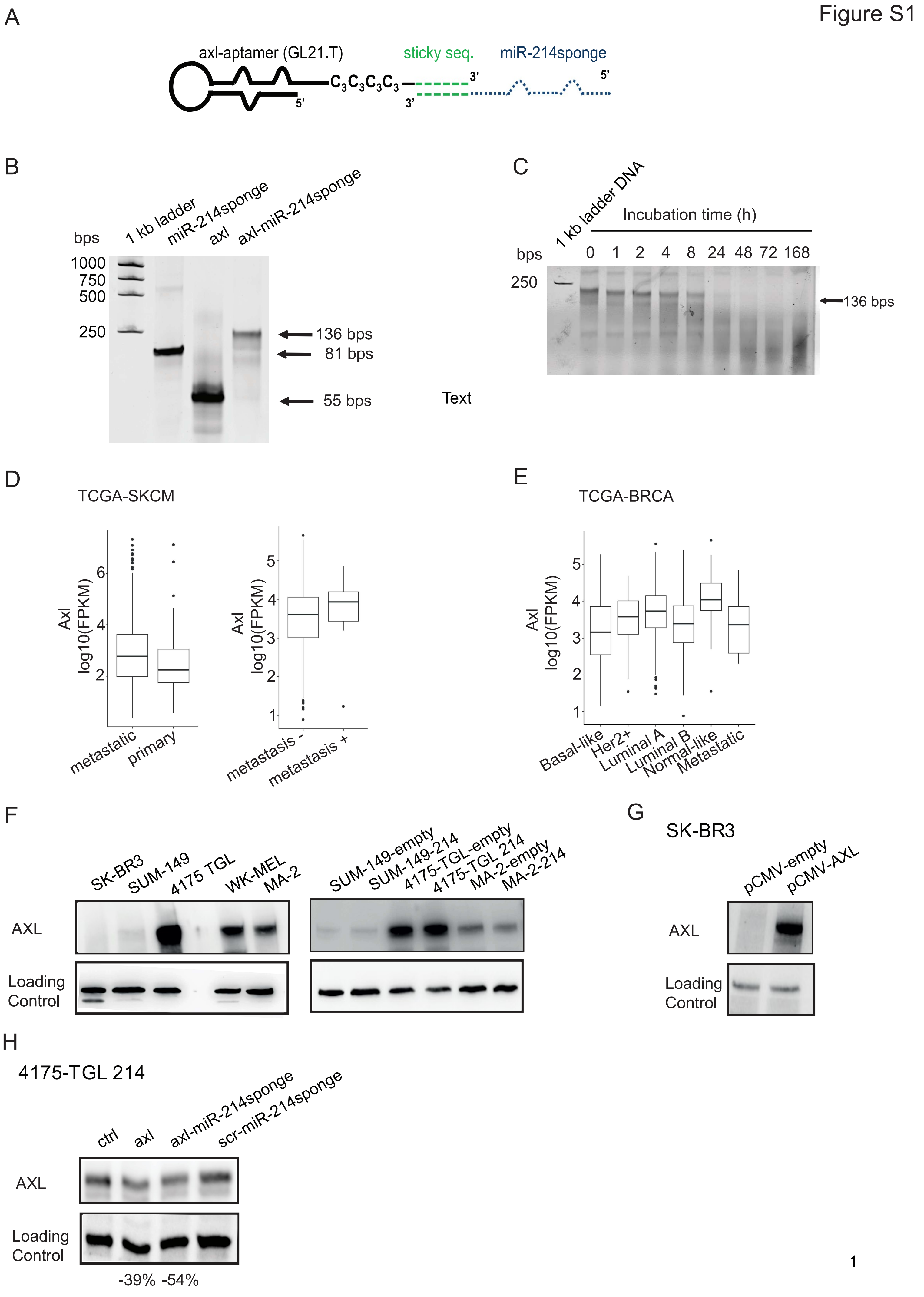
A
axl-aptamer (GL21.T)
sticky seq.
miR-214sponge
$C_3C_3C_3C_3$
3'
5'
5'
3'
B
bps
1 kb ladder
miR-214sponge
axl
axl-miR-214sponge
1000
750
500
250
136 bps
81 bps
55 bps
Text
C
1 kb ladder DNA
Incubation time (h)
bps
0 1 2 4 8 24 48 72 168
250
136 bps
D
TCGA-SKCM
Axl
log10(FPKM)
metastatic
primary
metastasis -
metastasis +
E
TCGA-BRCA
Axl
log10(FPKM)
Basal-like
Her2+
Luminal A
Luminal B
Normal-like
Metastatic
F
SK-BR3
SUM-149
4175 TGL
WK-MEL
MA-2
SUM-149-empty
SUM-149-214
4175-TGL-empty
4175-TGL 214
MA-2-empty
MA-2-214
AXL
Loading Control
G
SK-BR3
pCMV-empty
pCMV-AXL
AXL
Loading Control
H
4175-TGL 214
ctrl
axl
axl-miR-214sponge
scr-miR-214sponge
AXL
Loading Control
-39% -54%

Figure S1–Axl-miR-214sponge chimeric aptamer generation and axl expression. (A) Schematic representation of axl-miR-214sponge chimeric aptamer. (B) Representative polyacrylamide gel electrophoresis to verify the proper chimera formation. The 136 bps band corresponds to the axl-miR-214sponge conjugate, the 81 bps band shows the miR-214sponge fragment, the 55 bps coincides with the axl aptamer alone. (C) A representative picture of non-denaturing polyacrylamide gel electrophoresis showing the stability of axl-miR-214sponge chimeric aptamer (4 μM) in 80% human serum for the indicated time (hours=h). (D) axl expression (mRNA) in human metastatic (n=368) and primary (n=103) melanoma samples or in primary melanomas from patients with (n=3) or without (n=98) relapse (metastasis +/-) as obtained from TCGA-SKCM datasets. (E) axl expression (mRNA) in human Basal-like (n =190), Her2+ (n=82), Luminal A (n =562) or B (n =209), Normal-like (n =40) or metastatic (n =7) breast cancer samples as obtained from TCGA-BRCA datasets. TCGA data were downloaded through the TCGAbiolinks. Expression data are presented as log10(FPKM) with error bars representing standard deviations. (F) Western Blot (WB) analysis of axl expression in native or empty or miR-214 overexpressing (214) human breast cancer or melanoma SK-BR3, SUM-149, 4175-TGL, WK-MEL 214, MA-2 cell lines. GAPDH was used as loading control. (G) WB analysis for axl expression in SK-BR3 cells transfected with pCMV-AXL or pCMV-empty expression vectors. GAPDH was used as loading control. (H) WB analysis for axl expression in miR-214 overexpressing 4175-TGL cells following treatments with PBS control (ctrl) or axl or axl-miR-214sponge or scr-miR-214sponge (scramble) aptamer solutions for 48 hours. Protein modulations were calculated relative to ctrl and normalized on loading controls (vinculin) and expressed as percentages (%). (B-C) 1kb ladder DNA was used as referral for molecular weights. (B-H) Three independent experiments were performed, and representative results are shown. FPKM= Fragments Per Kilobase per Million mapped fragments.

Figure S2

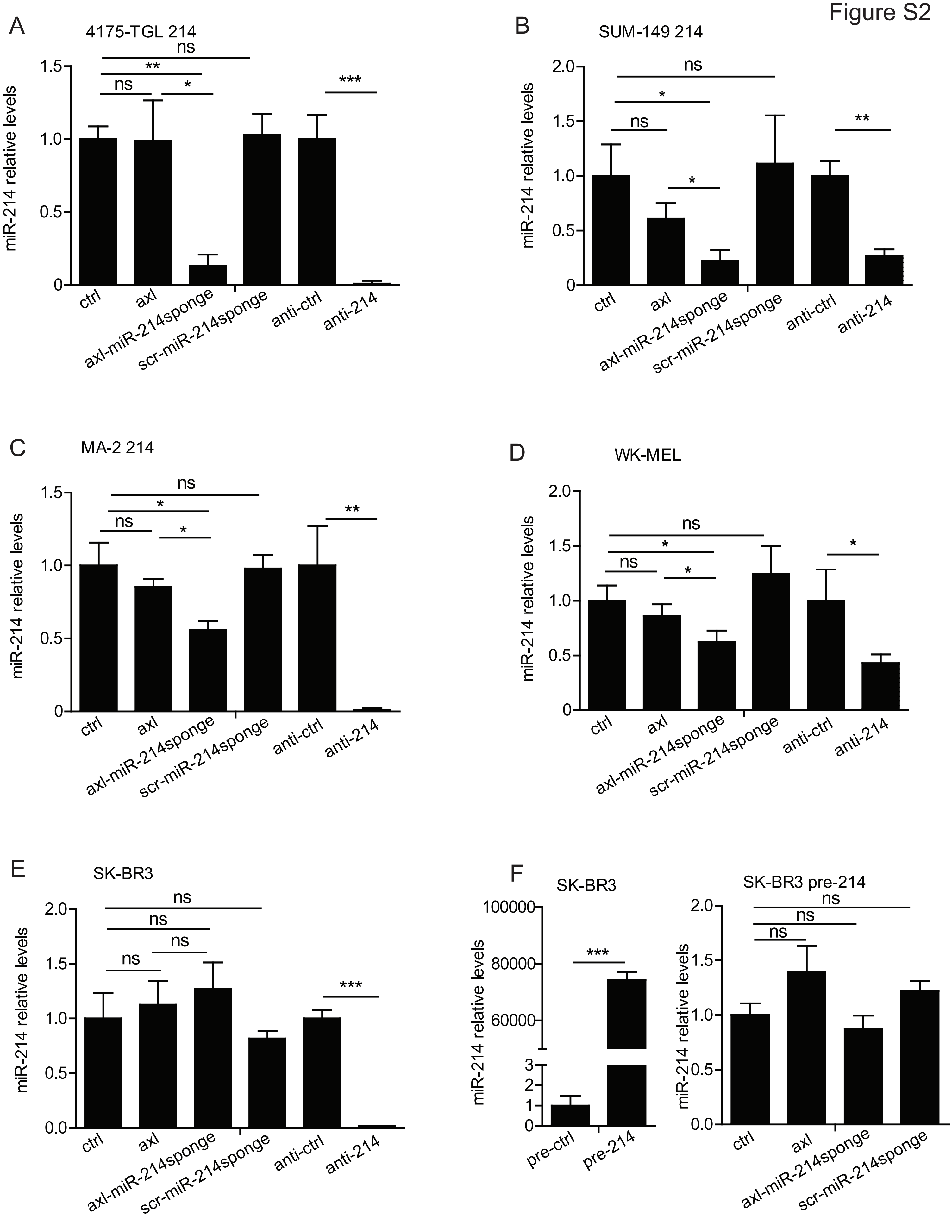
A
4175-TGL 214
miR-214 relative levels
1.5
1.0
0.5
0
ns
**
ns
*
***
ctrl
axl
axl-miR-214sponge
scr-miR-214sponge
anti-ctrl
anti-214
B
SUM-149 214
miR-214 relative levels
2.0
1.5
1.0
0.5
0
ns
*
ns
*
**
ctrl
axl
axl-miR-214sponge
scr-miR-214sponge
anti-ctrl
anti-214
C
MA-2 214
miR-214 relative levels
1.5
1.0
0.5
0
ns
*
ns
*
**
ctrl
axl
axl-miR-214sponge
scr-miR-214sponge
anti-ctrl
anti-214
D
WK-MEL
miR-214 relative levels
2.0
1.5
1.0
0.5
0
ns
*
ns
*
*
ctrl
axl
axl-miR-214sponge
scr-miR-214sponge
anti-ctrl
anti-214
E
SK-BR3
miR-214 relative levels
2.0
1.5
1.0
0.5
0.0
ns
ns
ns
ns
***
ctrl
axl
axl-miR-214sponge
scr-miR-214sponge
anti-ctrl
anti-214
F
SK-BR3
miR-214 relative levels
100000
80000
60000
3
2
1
0
***
pre-ctrl
pre-214
SK-BR3 pre-214
miR-214 relative levels
2.0
1.5
1.0
0.5
0.0
ns
ns
ns
ctrl
axl
axl-miR-214sponge
scr-miR-214sponge

Figure S2 – Axl-miR-214sponge chimeric aptamer affects miR-214 expression. (A-F) miR-214 relative expression levels for *axl*-positive miR-214 overexpressing 4175-TGL, SUM-149 or MA-2 cells or WK-MEL or *axl*-negative SK-BR3 cells after treatments with control (ctrl) solution, axl aptamer alone, axl-miR-214sponge or scr-miR-214sponge conjugates, evaluated by qRT-PCR analysis. Transfection with anti-miR-214 (anti-214, A-E) or pre-miR-214 (pre-214, F, left panel) or their relative controls (anti-ctrl or pre-ctrl) were also performed and miR-214 expression levels analyzed by qRT-PCR analysis. Results are shown as fold changes (mean ± SD) relative to controls, normalized on U44 or U6 small nucleolar RNA levels. Three independent experiments in triplicate were performed and a representative one is shown. ns= not significant, * $p < 0.05$, ** $p< 0.01$, *** $p< 0.001$. SD= Standard Deviation.

Figure S3

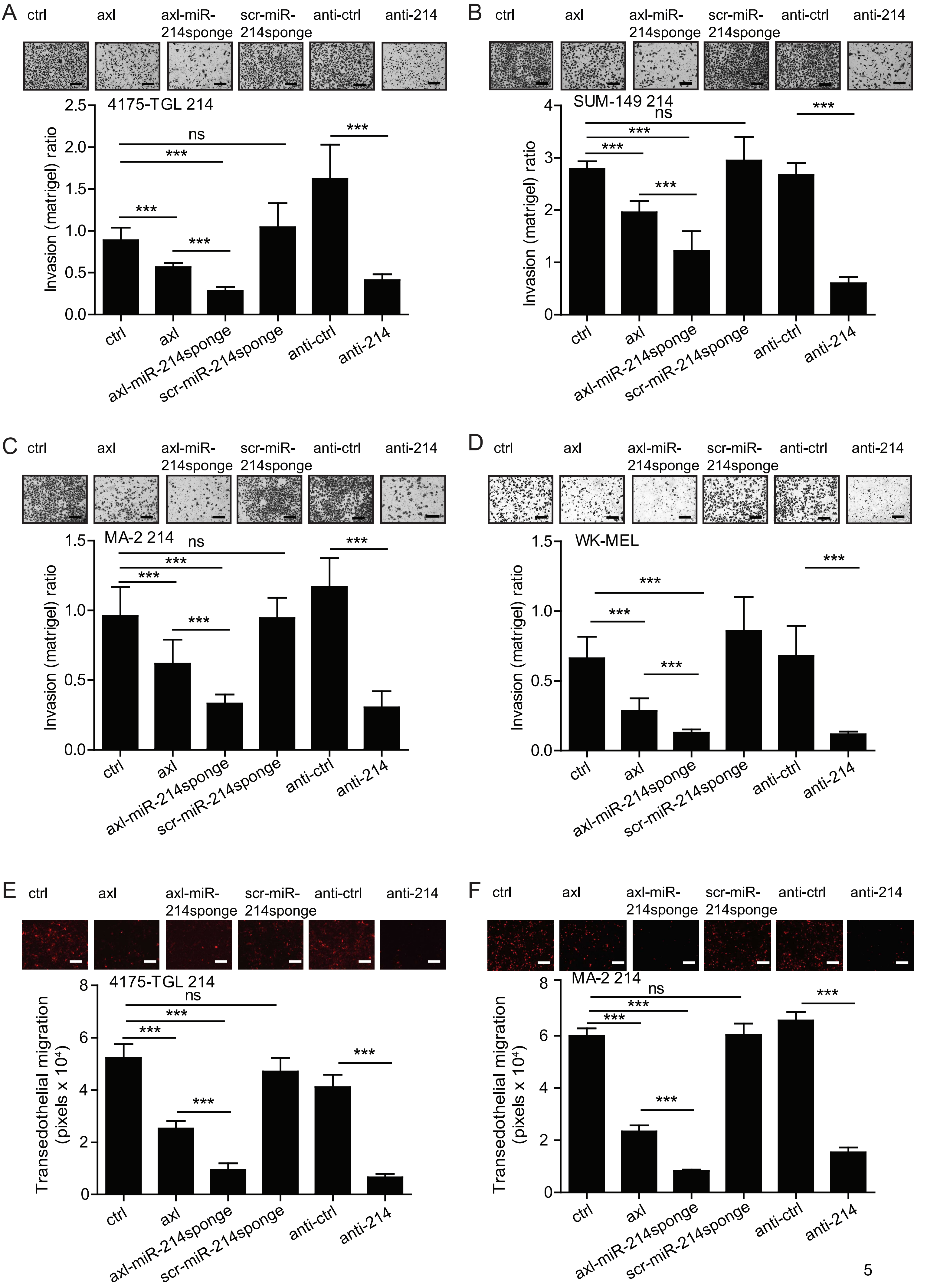
A
ctrl
axl
axl-miR-214sponge
scr-miR-214sponge
anti-ctrl
anti-214
4175-TGL 214
Invasion (matrigel) ratio
ns
***
B
SUM-149 214
C
MA-2 214
D
WK-MEL
E
4175-TGL 214
Transedothelial migration (pixels x 10⁴)
F
MA-2 214

Figure S3 - Axl-miR-214sponge chimeric aptamer impairs cancer cell invasion.

(A-F) Invasion through Matrigel (A-D) or through a HUVEC monolayer (E-F) assays for *axl*-positive miR-214 overexpressing 4175-TGL (A, E), SUM-149 (B), MA-2 (C, F) or not-engineered WK-MEL (D) cells treated with ctrl solution, axl aptamer alone or axl-miR-214sponge or scr-miR-214sponge conjugates. Transfections with anti-miR-214 (anti-214) and its relative control (anti-ctrl) were also performed. Top: representative photographs of invaded/transmigrated cells. Bottom: graphs of results expressed as (A-D) ratio of mean ± SEM of the area covered by invaded versus plated cancer cells or as (E-F) mean±SEM of the area (pixels) covered by transmigrated cells. At least two independent experiments (in triplicate) were performed and representative results are shown. ns = not significant; *** $p < 0.001$; SEM = Standard Error of Mean; scale bar = 50 μm.

Figure S4

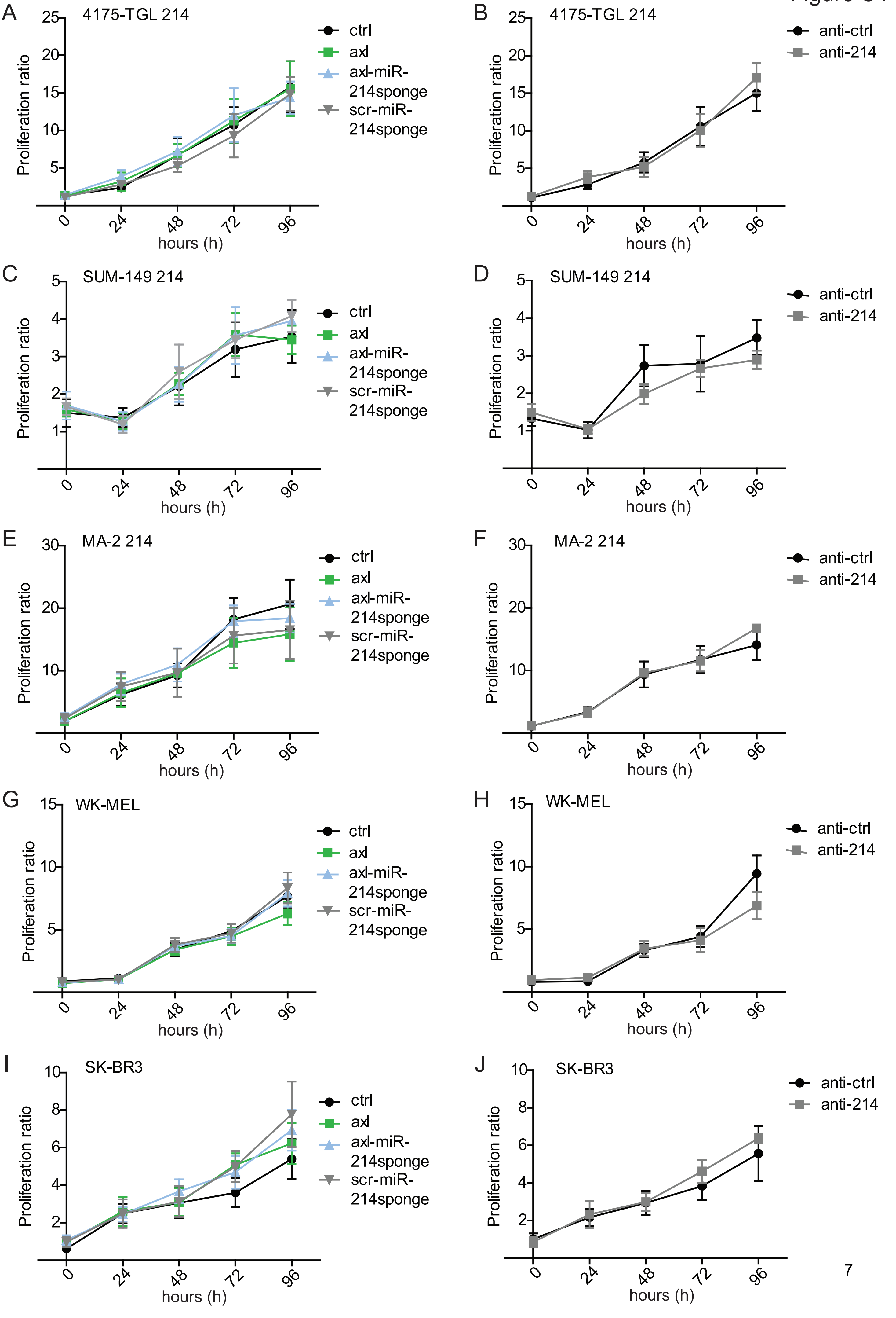
A
4175-TGL 214
Proliferation ratio
hours (h)
ctrl
axl
axl-miR-214sponge
scr-miR-214sponge
B
4175-TGL 214
anti-ctrl
anti-214
C
SUM-149 214
D
SUM-149 214
E
MA-2 214
F
MA-2 214
G
WK-MEL
H
WK-MEL
I
SK-BR3
J
SK-BR3

Figure S4– Axl-miR-214sponge chimeric aptamer does not affect *in vitro* proliferation. (A-J) Proliferation for *axl*-positive miR-214 overexpressing 4175-TGL (A-B), SUM-149 (C-D), MA-2 (E-F) or not-engineered WK-MEL (G-H) or *axl*-negative SK-BR3 (I-J) cells treated with ctrl solution, axl aptamer alone or axl-miR-214sponge or scr-miR-214sponge conjugates. Transfections with anti-miR-214 (anti-214) and its relative control (anti-ctrl) were also performed. Results are represented as mean±SD of the proliferation *ratio* versus plated cells, measured by optical density at 0-96h. At least 2 independent experiments (with triplicates) were performed and representative results are shown. SD = Standard Deviation.

Figure S5

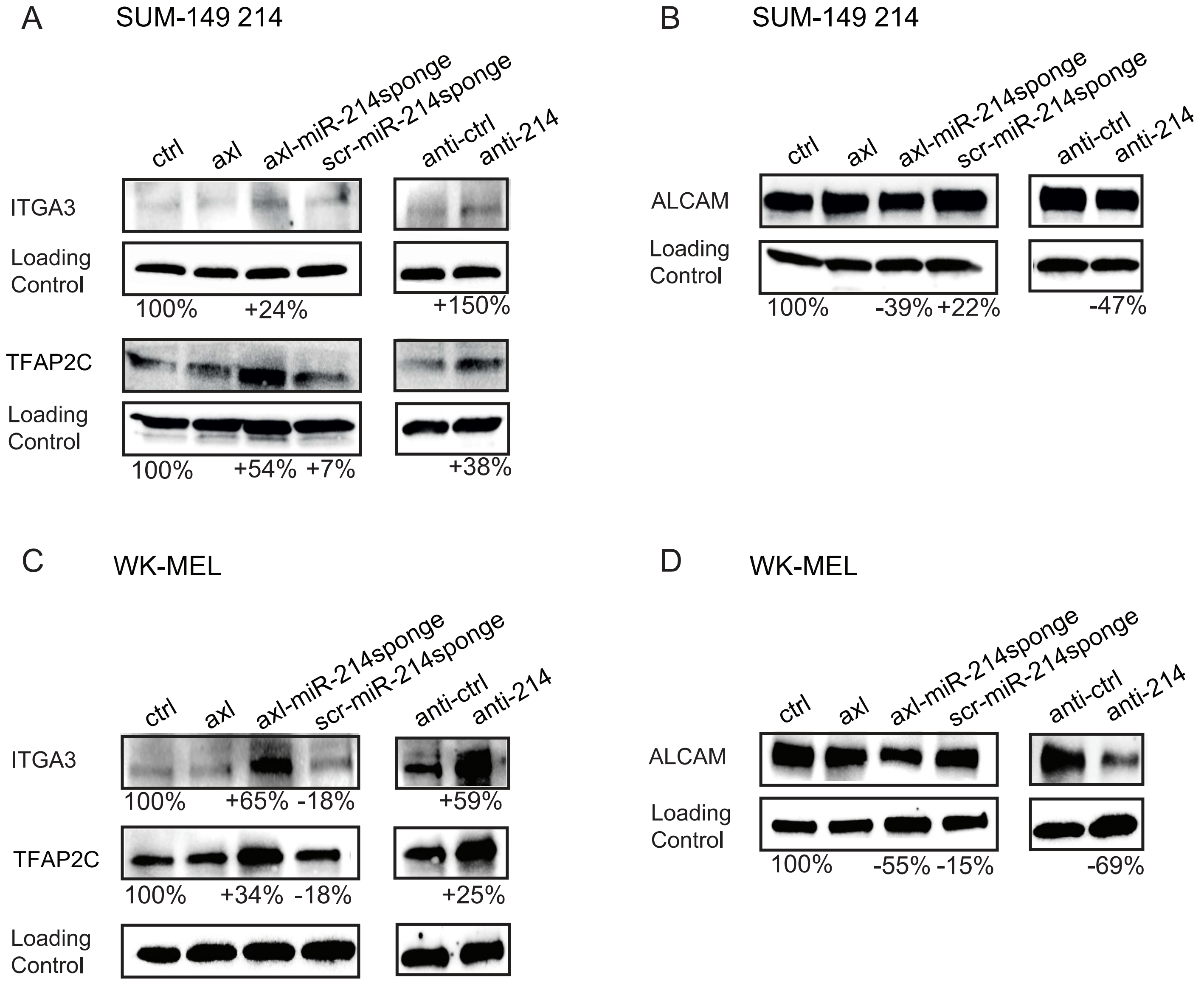
A
SUM-149 214
ctrl
axl
axl-miR-214sponge
scr-miR-214sponge
anti-ctrl
anti-214
ITGA3
Loading Control
100%
+24%
+150%
TFAP2C
Loading Control
100%
+54%
+7%
+38%
B
SUM-149 214
ctrl
axl
axl-miR-214sponge
scr-miR-214sponge
anti-ctrl
anti-214
ALCAM
Loading Control
100%
-39%
+22%
-47%
C
WK-MEL
ctrl
axl
axl-miR-214sponge
scr-miR-214sponge
anti-ctrl
anti-214
ITGA3
100%
+65%
-18%
+59%
TFAP2C
100%
+34%
-18%
+25%
Loading Control
D
WK-MEL
ctrl
axl
axl-miR-214sponge
scr-miR-214sponge
anti-ctrl
anti-214
ALCAM
Loading Control
100%
-55%
-15%
-69%

Figure S5– Axl-miR-214sponge chimeric aptamer affects miR-214 direct and indirect targets in *axl*-expressing cancer cells. (A-D) Western blot analysis of miR-214 direct targets ITGA3 and TFAP2C or indirect target ALCAM in *axl*-positive SUM-149 214 (A-B), WK-MEL (C-D) treated with ctrl solution, axl aptamer alone, axl-miR-214sponge or scr-miR-214sponge conjugates. Transfections with anti-miR-214 (anti-214) and its relative control (anti-control) were also performed. After 48 hours proteins were collected and protein modulations calculated relative to axl or anti-control and normalized on loading controls: hsp90 (A, top) or GAPDH (A, bottom; B; C, left; D, right) or vinculin (C, right) and expressed as percentages (%). At least three independent experiments were performed and representative results are shown.

Figure S6

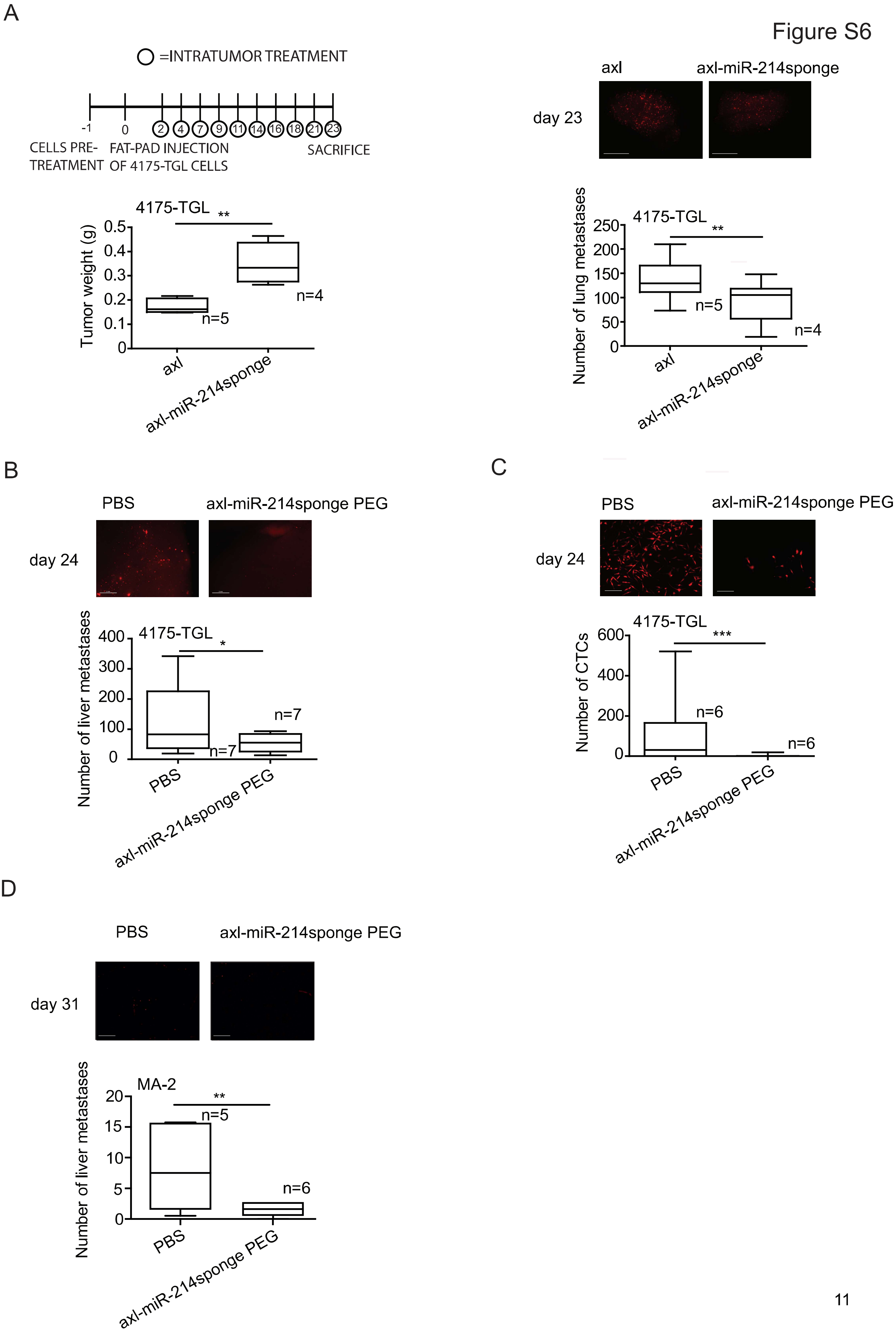
A
=INTRATUMOR TREATMENT
-1
0
2 4 7 9 11 14 16 18 21 23
CELLS PRE-TREATMENT
FAT-PAD INJECTION OF 4175-TGL CELLS
SACRIFICE
4175-TGL
**
Tumor weight (g)
0.5
0.4
0.3
0.2
0.1
0
n=5
n=4
axl
axl-miR-214sponge
axl
axl-miR-214sponge
day 23
Number of lung metastases
250
200
150
100
50
0
4175-TGL
**
n=5
n=4
axl
axl-miR-214sponge
B
PBS
axl-miR-214sponge PEG
day 24
Number of liver metastases
400
300
200
100
0
4175-TGL
*
n=7
n=7
PBS
axl-miR-214sponge PEG
C
PBS
axl-miR-214sponge PEG
day 24
Number of CTCs
600
400
200
0
4175-TGL
***
n=6
n=6
PBS
axl-miR-214sponge PEG
D
PBS
axl-miR-214sponge PEG
day 31
Number of liver metastases
20
15
10
5
0
MA-2
**
n=5
n=6
PBS
axl-miR-214sponge PEG

Figure S6 –Axl-miR-214sponge chimeric aptamer intratumor or systemic treatments reduce breast cancer dissemination in mice. (A) Scheme of the experiment: Red Fluorescent Protein (RFP)-expressing 4175-TGL cells pretreated with axl or axl-miR-214sponge aptamers were injected into the mammary gland fat pad of NOD/SCID/IL2R null mice. Then, axl or axl-miR-214sponge aptamers were administered into the tumors starting from 2 days post-injection (3 treatments/week, 300 pmol in 100 µl, 10 injections in total, as indicated) and primary tumor weight (grams = g) and total number of lung metastases (mean±SEM) were analyzed 23 days post-tumor-cell injections for the indicated number (n) of mice. (B-D) Number of liver metastases (B, D) and Circulating Tumor Cells (CTCs) (C) referring to the experiments presented in Figure 4A-B. Representative images of fluorescent lung or liver metastases or CTCs are shown. ** $p < 0.01$; SEM = Standard Error of Mean; Scale bar=800 µm.

Figure S7

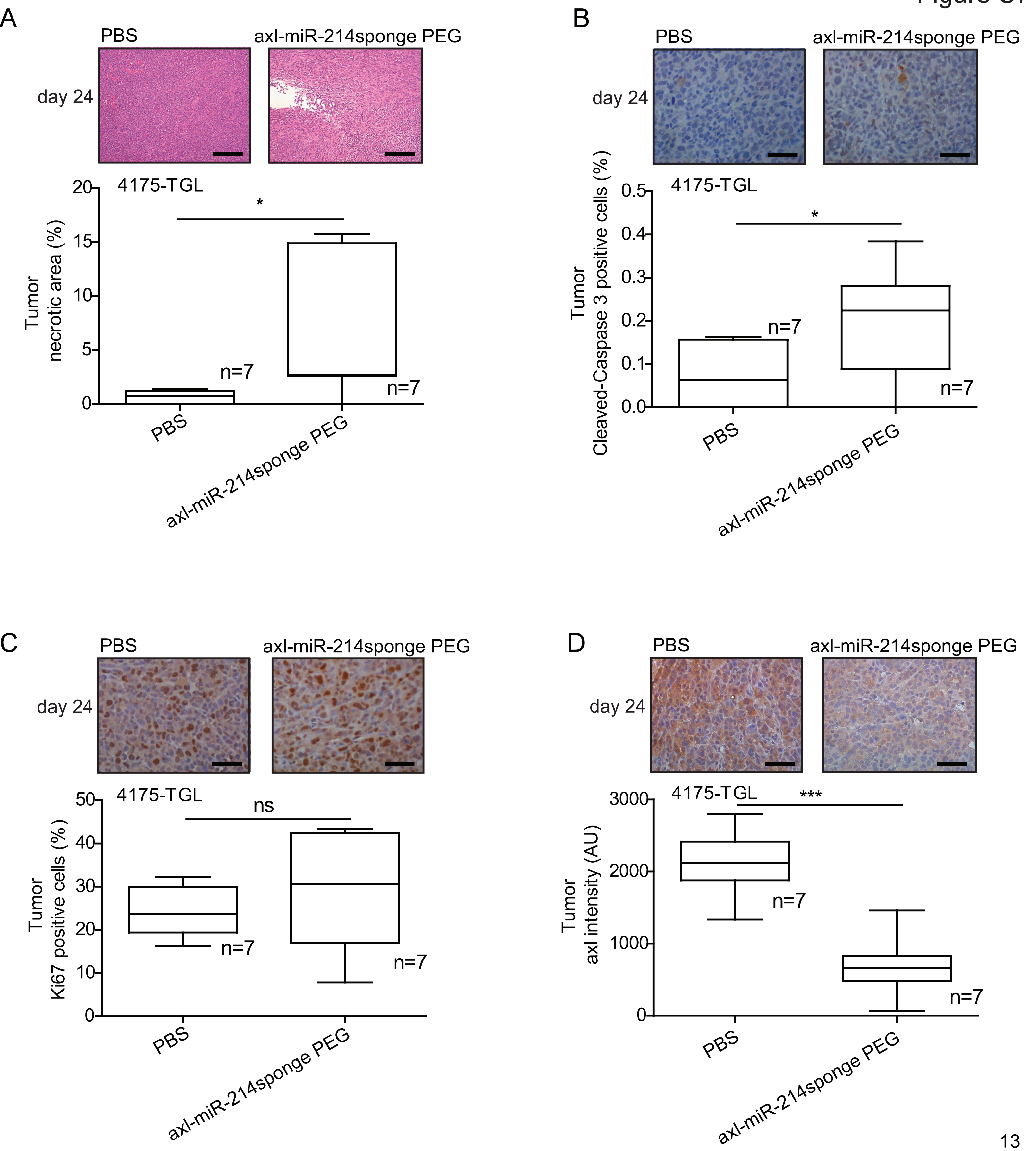

A
PBS
axl-miR-214sponge PEG
day 24
4175-TGL
Tumor necrotic area (%)
20
15
10
5
0
*
n=7
n=7
PBS
axl-miR-214sponge PEG
B
PBS
axl-miR-214sponge PEG
day 24
4175-TGL
Tumor Cleaved-Caspase 3 positive cells (%)
0.5
0.4
0.3
0.2
0.1
0.0
*
n=7
n=7
PBS
axl-miR-214sponge PEG
C
PBS
axl-miR-214sponge PEG
day 24
4175-TGL
Tumor Ki67 positive cells (%)
50
40
30
20
10
0
ns
n=7
n=7
PBS
axl-miR-214sponge PEG
D
PBS
axl-miR-214sponge PEG
day 24
4175-TGL
Tumor axl intensity (AU)
3000
2000
1000
0
***
n=7
n=7
PBS
axl-miR-214sponge PEG

Figure S7 - Axl-miR-214sponge chimeric aptamer induces necrosis and apoptosis in breast cancer xenotransplants. (A-D) FFPE sections of 4175-TGL cell-derived primary tumors treated as in Figure 4B were stained with (A) H&E and necrotic areas evaluated: representative images are shown on top of plots presenting the percentage (%) of necrotic versus total areas shown as mean±SEM for the indicated number (n) of mice (10 fields/each mouse). Primary tumors were also stained for (B) Cleaved Caspase-3, (C) Ki67 or (D) *axl* and nuclei were counterstained with Hematoxylin (blue). Representative pictures are shown on top of plots presenting the percentage (%) of positive versus total cells (A-C) or staining intensity (D) shown as mean±SEM for the indicated number (n) of mice (10 fields/each mouse). IHC = immunohistochemistry; H&E = Hematoxilyin & Eosin; FFPE: Formalin-Fixed, Paraffin Embedded; ns = not significant; * $p < 0.05$; *** $p < 0.001$; SEM = Standard Error of Mean; scale bar = 100 µm (A) or 25 µm (B-D).

Figure S8

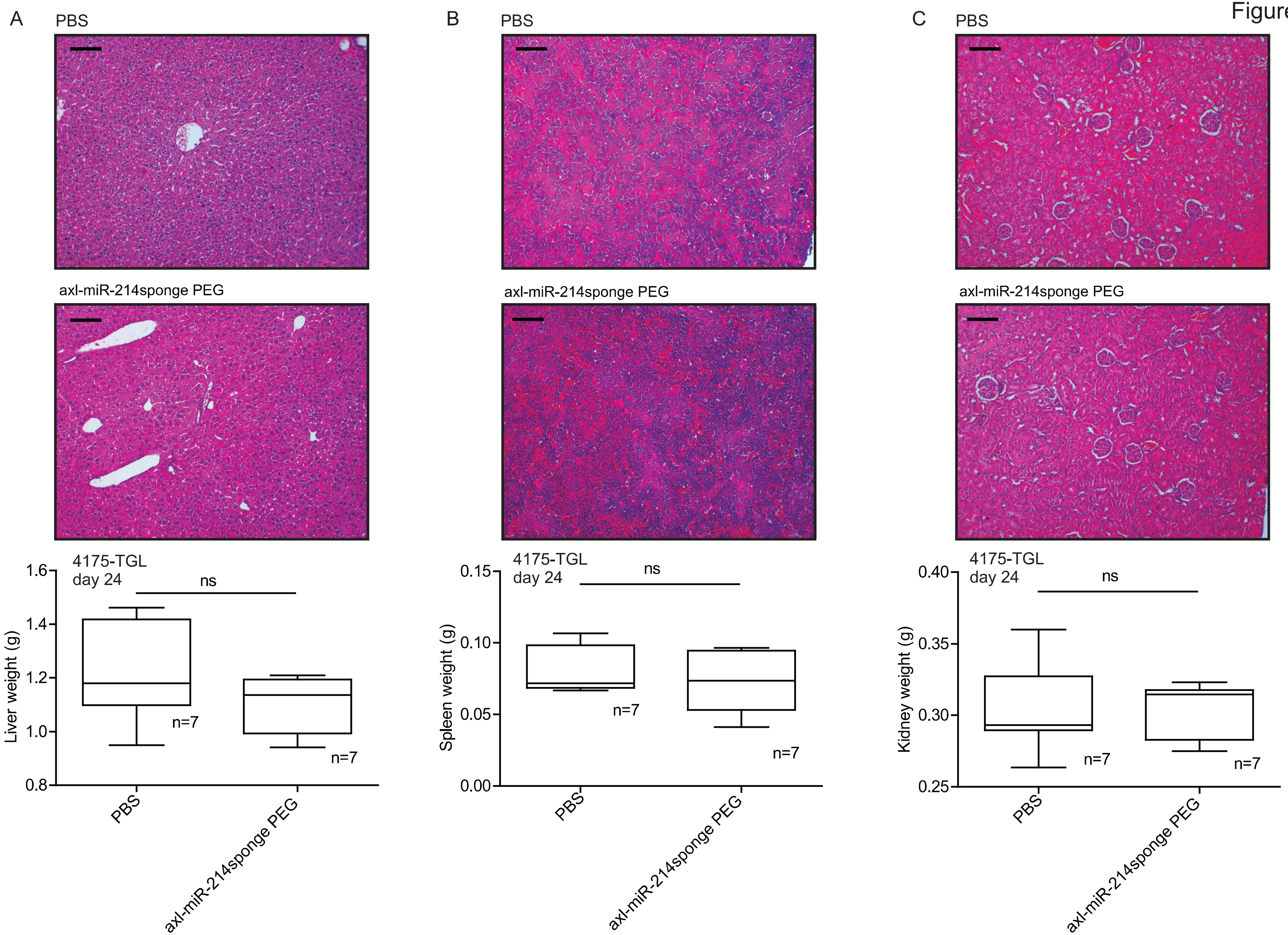
A
PBS
axl-miR-214sponge PEG
4175-TGL
day 24
ns
Liver weight (g)
1.6
1.4
1.2
1.0
0.8
n=7
n=7
PBS
axl-miR-214sponge PEG
B
PBS
axl-miR-214sponge PEG
4175-TGL
day 24
ns
Spleen weight (g)
0.15
0.10
0.05
0.00
n=7
n=7
PBS
axl-miR-214sponge PEG
C
PBS
axl-miR-214sponge PEG
4175-TGL
day 24
ns
Kidney weight (g)
0.40
0.35
0.30
0.25
n=7
n=7
PBS
axl-miR-214sponge PEG

Figure S8– Axl-miR-214sponge chimeric aptamer is not toxic for mice when delivered systemically. (A-C) Mice were injected with Red Fluorescent Protein (RFP)-expressing 4175-TGL cells and treated as in Figure 4B. The weight of liver (A), spleen (B) and kidneys (C) was evaluated at day 24 (final point) and sections stained with H&E. Representative pictures of organs (scale bar=100 μm) are shown on top of plots representing the mean±SEM of weights for the indicated number (n) of mice. ns = not significant; SEM= Standard Error of Mean; H&E = Hematoxilyn & Eosin; g = grams.
